# Identification and structural characterization of LytU, a unique peptidoglycan endopeptidase from the lysostaphin family

**DOI:** 10.1038/s41598-017-06135-w

**Published:** 2017-07-20

**Authors:** Vytas Raulinaitis, Helena Tossavainen, Olli Aitio, Jarmo T. Juuti, Keiichi Hiramatsu, Vesa Kontinen, Perttu Permi

**Affiliations:** 1 Program in Structural Biology and Biophysics, Institute of Biotechnology, University of Helsinki, Viikinkaari 1, P.O. Box 65, FI-00014 Helsinki, Finland; 2 Antimicrobial Resistance Unit, Department of Infectious Disease Surveillance and Control, National Institute for Health and Welfare, P.O. Box 30, FI-00271 Helsinki, Finland; 3 Research Centre for Infection Control Science, Juntendo University, Bunkyo-ku, Tokyo Japan; 4 Department of Biological and Environmental Science, and Department of Chemistry, Nanoscience Center, University of Jyvaskyla, P.O. Box 35, FI-40014 Jyvaskyla, Finland

## Abstract

We introduce LytU, a short member of the lysostaphin family of zinc-dependent pentaglycine endopeptidases. It is a potential antimicrobial agent for *S. aureus* infections and its gene transcription is highly upregulated upon antibiotic treatments along with other genes involved in cell wall synthesis. We found this enzyme to be responsible for the opening of the cell wall peptidoglycan layer during cell divisions in *S. aureus*. LytU is anchored in the plasma membrane with the active part residing in the periplasmic space. It has a unique Ile/Lys insertion at position 151 that resides in the catalytic site-neighbouring loop and is vital for the enzymatic activity but not affecting the overall structure common to the lysostaphin family. Purified LytU lyses *S. aureus* cells and cleaves pentaglycine, a reaction conveniently monitored by NMR spectroscopy. Substituting the cofactor zinc ion with a copper or cobalt ion remarkably increases the rate of pentaglycine cleavage. NMR and isothermal titration calorimetry further reveal that, uniquely for its family, LytU is able to bind a second zinc ion which is coordinated by catalytic histidines and is therefore inhibitory. The pH-dependence and high affinity of binding carry further physiological implications.

## Introduction

The emergence and spread of new drug-resistant *Staphylococcus aureus* strains are a growing global concern, particularly in hospital settings^1^. The primary and key defence mechanism of the pathogen is to increase the number of peptidoglycan (PG) layers in its cell wall (CW)^2^. Selective pressure driving this change results from the extensive use of the most prominent antistaphylococcal agents, beta-lactam antibiotics (e.g. methicillin) and glycopeptides (e.g. vancomycin) causing these antibiotics to become increasingly inefficient in combat against the new treatment-resistant strains^3^. The mechanism of action for these drugs is to interfere with the synthesis of *S. aureus* CW, i.e. its PG. Accordingly, targeting PG for lysis can serve antimicrobial function alone or in combination with other therapeutic approaches.

The target of this study, the sa0205 coding frame of the *S. aureus* genome, was identified as one of the highly induced genes in cultures treated with the aforementioned antibiotics^4^. It was shown to be under the control of the VraSR two-component system that regulates genes involved in CW peptidoglycan synthesis in *S. aureus* ^5^. Interestingly, transcription of *sa0205* is upregulated 50- and 25-fold by cationic antimicrobial peptides and vancomycin, respectively. However, based on the amino acid sequence, the product of this coding frame was not a PG synthesizing enzyme but a putative lysostaphin family endopeptidase that hydrolyses PG.

Lysostaphin family endopeptidases are zinc-dependent enzymes that cleave pentaglycine interpeptide bridges of CW PG. They are utilized as autolysins or weapons against competing strains. Indeed, promising results on the use of lysostaphin as an antimicrobial agent have been reported^6–10^. Lysostaphin itself is secreted by *Staphylococcus simulans* biovar *staphylolyticus* and targets the CW of other staphylococci with its SH3b domain. Conjugation of this domain with a catalytic domain of *S. aureus* autolysin LytM produced chimeras that may supersede the efficacy of lysostaphin in their medical applications^11, 12^.

The catalytic mechanism of the lysostaphin family enzymes has recently been investigated^13^. It involves a zinc ion which polarizes the scissile peptide bond by coordinating the carbonyl oxygen, and two catalytic histidines which activate a water molecule or a hydroxide ion to act as a nucleophile to attack the carbonyl carbon. Involvement of a previously unacknowledged tyrosine residue as a hydrogen bond donor to the transition state was revealed. Although lysostaphin family members are widely regarded as zinc-dependent enzymes, a partial activity of LytM can be restored by Co^2+^, Mn^2+^ and Cu^2+^ ions^14^. The *in vitro* active pH range for both LytM and lysostaphin is 5–9^14–16^ and the optimum for lysostaphin is 7.5^16^.

Among the factors hampering investigations of lysostaphin family enzymes is the insolubility of their substrate, PG. Several alternative substrates and approaches have been proposed for activity assessment^14, 17–19^. None of these substrates has become commonly utilized and the primary analysis of activity remains the lysis of whole cells, which provides multiple variables and obstacles to study structure and catalysis. Furthermore, *in situ* studies are complicated due to the presence of other autolytic enzymes that are potentially able to compensate for their counterparts and thus a comprehensive registry of autolysins is necessary. Finally, the regulation of autolytic activity remains unclear and so far its cornerstone has been activation by cleavage of protoenzymes^17, 20, 21^.

Our study brings new insight into the lysostaphin family by introducing the product of the gene *sa0205*. Consistently with the present nomenclature, we call it LytU. Here we present solution structures of its catalytic domain and demonstrate the tight binding of a second zinc ion, which effectively inhibits the enzyme. The physiologically relevant affinity and pH sensitivity of this binding are pertinent to a regulation of catalytic activity. We examine catalysis of substrate and metal ion effect by monitoring pentaglycine cleavage by NMR.

## Results

### LytU is a member of the lysostaphin family

**Figure 1:**
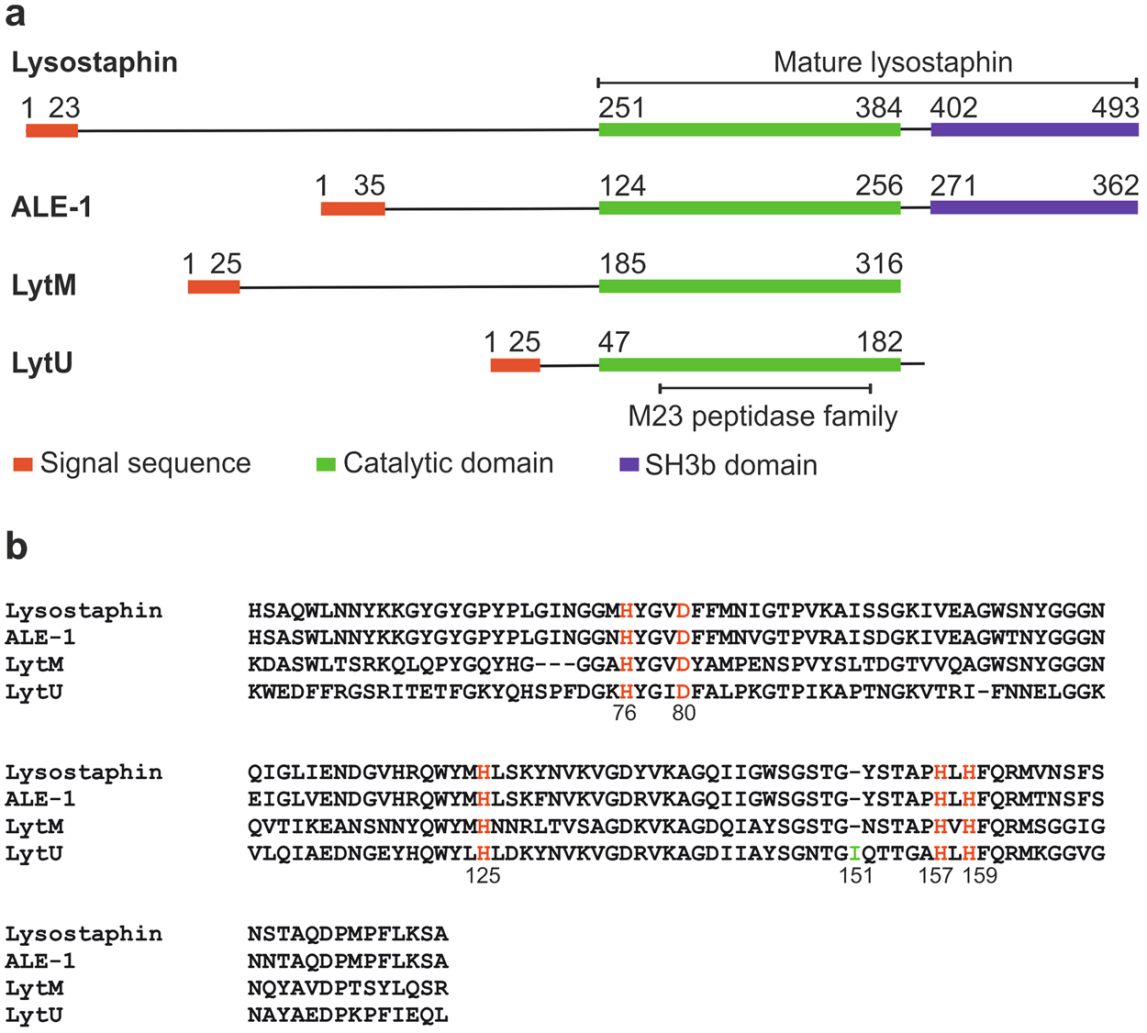
Comparison of M23 endopeptidase amino acid sequences. (**a**) Comparison of full-length lysostaphin, ALE-1, LytM, and LytU. N-termini of all proteins begin with a signal sequence that directs them to the cellular membrane and outward of the cytoplasm. The enzymes have unique N-domains (those for ALE-1 and lysostaphin are analogous, consisting of tandem 13 amino acid length repeats), for which functions remain unknown. The N-domain may need to be cleaved off for the enzymatic activity of catalytic domain (as for LytM, the activating *in vivo* enzyme is unknown^20^), need not be cleaved off (ALE-1^17^), or the enzyme may increase activity upon cleavage (4.5-fold for lysostaphin^21^). Lysostaphin and ALE-1 possess, while autolytic LytM lacks SH3b domain downstream their catalytic domains to target extracellular substrates^20, 42, 43^. Structural predictions for LytU were made using SignalP^44^, HMMTOP^45^ and MODELLER^46^. Signal sequences are marked as indicated in the UniProt database, except for LytU sequence, which was predicted using SignalP^44^. (**b**) Alignment of the catalytic domains with ClustalW^47^. Conserved residues involved in zinc ion coordination (H76, D80 and H159) and catalytic (H125 and H157) are marked in red. The LytU unique insertion is highlighted in green and can be lysine in some *S. aureus* species.

To retrieve information regarding the structural organization of LytU, we carried out an analysis of LytU primary structure and compared it to lysostaphin family members. Residues 7–25 of LytU are predicted to form a transmembrane helix followed by a short, disordered region (26–48) and a catalytic domain homologous to lysostaphin (i.e. LytM domain, Fig. 1a). Within this region, residues 74–167 contain the M23 family peptidase domain (Fig. 1a). Residues H76, D80, and H159 in LytU are expected to coordinate a zinc ion (Fig. 1b) and H125 and H157 to cleave peptide bonds between consecutive glycines^22, 23^. LytU lacks the CW targeting SH3b domain present in lysostaphin. A unique sequence feature of LytU is the insertion at residue position 151 in the otherwise conserved region. Among the different *S. aureus* subsp. *aureus* strains this residue is either isoleucine or lysine.

### LytU affects cell division

**Figure 2:**
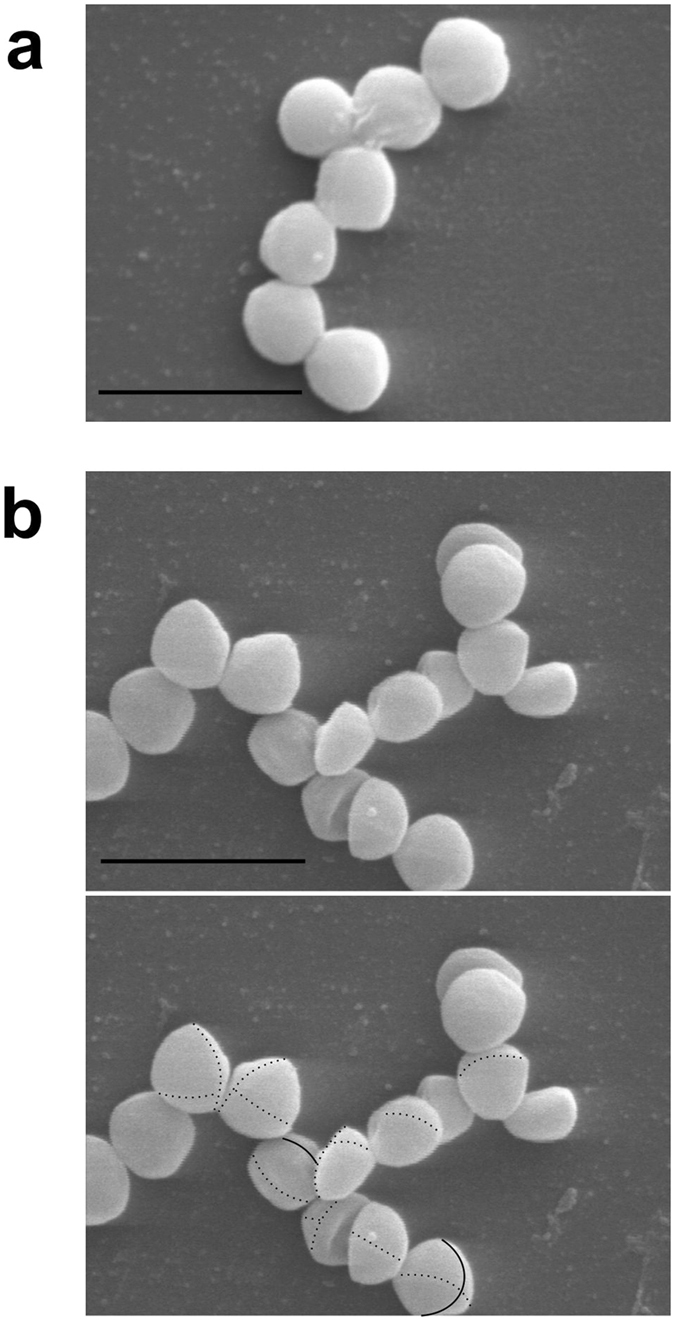
Phenotype of the LytU deficient mutant. (**a**) Scanning electron micrograph of the wild-type *S. aureus* Newman control. Scale bar, 2 µm. **(b)** On top an image of LytU-deficient Newman strain mutant cells without markings and on the bottom the same picture with markings. Scale bar, 2 µm. Sites of active septa are marked with thin solid lines while the previous cell division line and the one before that are marked with thin dashed lines. While sites of cell division (septa) and daughter cells filling up to spheres were abundantly present in the wild type sample as well as in the mutant sample it was very hard to find any evidence on the location of the previous cell division planes in the control sample. For the mutant strain sample adjusting focus and magnification in EM made tracking of the traces of the previous cell division sites easier than what can be visualised in any still photograph. It should be noted that the position of the next cell division plane in *S. aureus* is independent of the previous one. Therefore, not every cell in the frame has the latest division circle, or a part of the one before, detectable on the front surface (possible area of detection being less than half of the actual full cell surface). Transmission EM study did not reveal any differences between the wild type and LytU mutant and the pictures are thus not shown.

To find a functional role for LytU in *S. aureus* lifecycle, we started by removing the gene from the genome. Deletion of LytU gene did not affect the bacterial growth of liquid or plate cultures indicating that LytU is not essential for *S. aureus* survival. However, when we examined *S*. *aureus* cells using scanning electron microscopy (EM) a small difference in the CW was found (Fig. 2). On the surface of ΔLytU cells, traces of the previous cell division plane could still be seen even though the next division was already on-going. The observed structure resembles the tearing edge of a paper sheet and the finding suggested that LytU is directly involved in the opening of the CW upon cell division.

**Figure 3:**
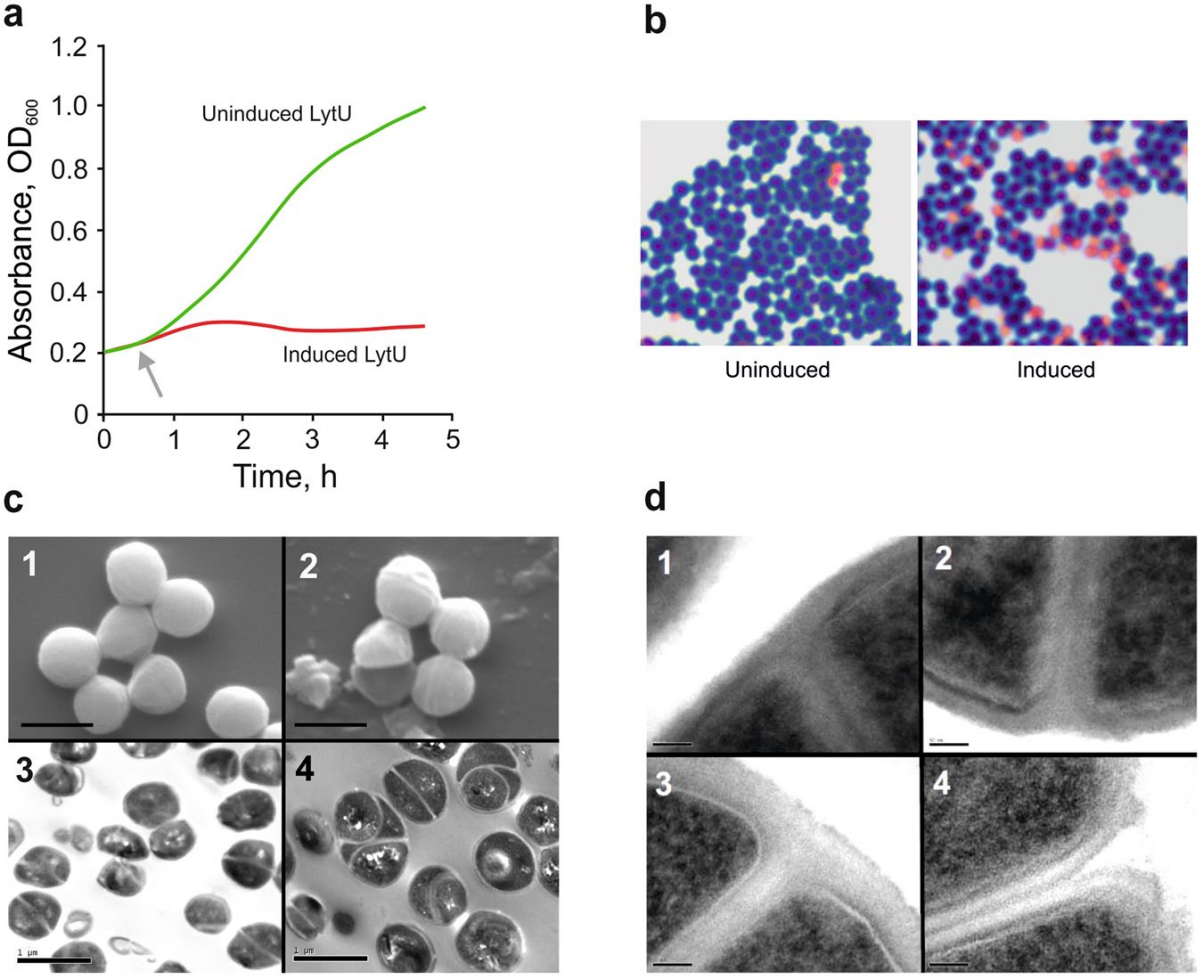
Overexpression of LytU. (**a)** The growth of *S. aureus* Newman strain containing an expression plasmid producing LytU. An immediate arrest of growth upon induction as compared to the uninduced culture was seen. These cultures were sampled for microscopic examinations. **(b)** Light microscope images of Gram-stained samples from a repeat for the one shown in panel (a) at the end time of 2 h 35 min when cultures were sampled for EM. A significant increase in a number of dead cells is seen in the induced culture. **(c)** Scanning EM and transmission EM study of the uninduced (1 and 3, respectively) and induced (2 and 4, respectively) samples. Scale bars, 1 µm. Images of the uninduced sample did not reveal any differences from normal growth thus excluding any defects caused by the presence of the expression vector or the uninduced insert. In scanning EM images, dead empty cells and cell debris are seen, explaining the stickiness of the sample and in accordance with Gram-staining results shown in panel (b). Multiple cell division sites are seen on the surface of the cells and transmission EM pictures clearly showed multiple daughter cells (up to about three divisions in maximum) within one mother cell. **(d)** Close-ups of the site of daughter cell separation. Scale bars, 50 nm. Image 1, uninduced cell are showing normal early-stage septa (plasma membranes formed) at the onset of CW synthesis. These kinds of divisions were very hard to find in the induced sample. Image 2, the later stage of a normal (uninduced) cell where the plasma membranes of daughter cells have started to separate and CW PG is accumulating in between. Normal progression inwards curving and restructuring of the CW surface that forms a surface groove is seen. Image 3, a cell where LytU has been induced. The CW between daughter cells is well formed, but no separation or reformation of mother cell’s CW is still seen. Image 4, the eventually broken CW of a LytU overexpressing cell in which the PG layer has not been restructured.

Contrary to deletion of the LytU gene, its overexpression had a clear effect on the growth of *S. aureus* cells. The growth was arrested upon induction and a subsequent increase of cell lysis in the culture was observed (Fig. 3a,b). We further examined the cultures using EM, which revealed that while cell division for individual cells progressed uninterrupted and completed normally, daughter cell separation was blocked (Fig. 3c). Eventually, after a few divisions, the CW ruptured under the expansion pressure (Fig. 3d) thereby causing the increase of broken cells in the culture. This was observed as an increased stickiness of the EM samples and a noticeable presence of cell debris. The results suggest that excess of LytU impaired the mechanisms involved in the opening of the CW upon cell division.

### LytU is anchored in the plasma membrane and its soluble part is extracytoplasmic

**Figure 4:**
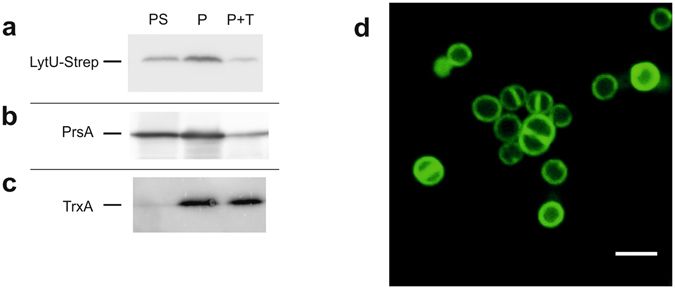
Determination of orientation and localisation of LytU in the plasma membrane. (**a, b, c**) Trypsin treatment of protoplast surface proteins showing that LytU behaves like a protein outside on the plasma membrane surface. The three vertical immunoblotting lanes in the assay correspond to: **P**, protoplasts; **PS**, protoplast supernatant; **P + T**, protoplasts treated with 1 mg ml^-1^ trypsin. As the level of production from the used expression vector is controlled by the amount of xylose inducer, 0.02% xylose was used to get very low amounts of LytU, resembling the natural conditions. The controls are shown in Supplementary Fig. S1. (**d**) Confocal microscopy image of *S. aureus* cells showing fluorescence of the N-terminal GFP-fusion to LytU protein. Scale bar, 2 μm. Clear localization of the fusion protein in the plasma membrane is seen as well as some possible accumulation at cell division sites (see text and Methods for details).

Fractionation experiment with the overexpressing strain showed that LytU is dominantly saturated in the cellular fraction containing the membrane. The orientation of the protein in the plasma membrane was then demonstrated by immunodetection of the C-terminal Strep-tag (Fig. 4). LytU-Strep was detected in whole cells and protoplasts. In addition, a significant amount of LytU-Strep was also present in the protoplast supernatant that contains the CW (Fig. 4a). Trypsin treatment degraded a major proportion of LytU-Strep in the protoplasts, indicating that the hydrophilic domain of LytU is on the outer surface of the membrane. The PrsA (protein secretion A) control, which is a known lipoprotein with a large extracytoplasmic domain^24^, behaved in a similar manner (Fig. 4b). Only traces of TrxA (thioredoxin A), which is a known cytoplasmic protein^25^, were detected in the protoplast supernatant, indicating that protoplast lysis was minimal and TrxA was not degraded in the trypsin treatment (Fig. 4c). The assay controls are shown in Supplementary Fig. S1.

To examine LytU distribution in the plasma membrane, we employed fluorescent confocal microscopy to observe the localization pattern of the GFPuv4-LytU fusion protein in exponentially growing *S. aureus* RN4220 (Fig. 4d). We observed the fluorescent protein only in the membrane, which is in good agreement with the results of our cell fractionation experiment. Overexpressed GFPuv4-LytU was distributed all over the cell membrane but higher fluorescence in septa could be seen as can be expected due to the presence of a double membrane and an enhancement effect of accumulated fluorescence. Interestingly, the slightly increased intensity at the sites where a new cell division plane is forming may suggest that at least part of the overexpressed GFPuv4-LytU could accumulate into the site of the next cell division.

### LytU lyses *S. aureus* cells

We subjected whole cells from actively growing and late stationary *S. aureus* cultures to autolysis. These data indicated that, under the examined conditions, old cells do not significantly undergo autolysis while young cells do so (Supplementary Fig. S2). Next, in order to study the effect of LytU on *S. aureus* cells, we added purified recombinant LytU to *S. aureus* cell cultures. Intriguingly, we observed that LytU effectively lysed both cell types and that the effect was additive to the activity originating from the cells. EDTA chelation prevented autolysis in all cases indicating that the process is cation dependent. The effect of cations on cell lysis was similar in both old and young cells suggesting that cell components participating in LytU-stimulated lysis were similar as well.

### LytU cleaves pentaglycine

**Figure 5:**
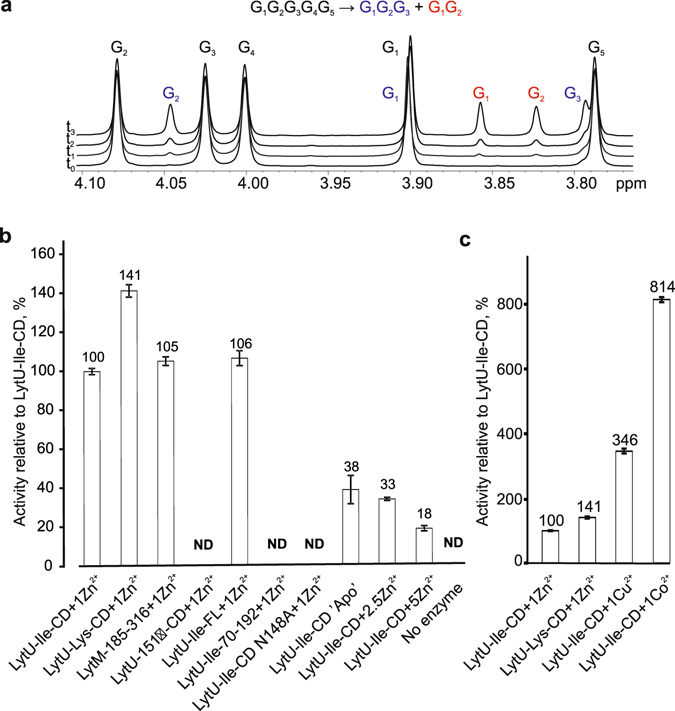
Catalytic activity. (**a**) Representative ^1^H spectra of 2 mM pentaglycine in PBS buffer (pH 7.3, 90% D_2_O) before (t_0_) and during the cleavage by LytU (t_1_–t_3_). Pentaglycine is cleaved into tri- and diglycine. Substrate and product signals are labelled. Pentaglycine and product G1 signals overlap. Integration of product signals as a percent of TSP standard (not shown) allows to monitor and quantify the reaction rate. **(b** and **c)** Relative activity of proteins as a percentage of the activity of one zinc-bound catalytic domain of LytU-Ile. Reactions were linear for up to 70 hours. CD, catalytic domain, FL, full-length, ND, not detected. The bars represent standard error (SEM) of at least two independent measurements.

We established the catalytic activity of LytU directly by monitoring pentaglycine cleavage by ^1^H NMR spectroscopy. This approach is direct, easily quantifiable, reproducible and real-time. A typical course of the reaction is shown in Fig. 5a. Identification of substrate and product peaks with ^1^H, ^13^C HMBC spectra revealed that LytU hydrolyses pentaglycine into di- and triglycine (Supplementary Fig. S3).

There was no significant difference in substrate cleavage between the full-length soluble LytU (residues 26–192) and LytU(49–192) that includes the catalytic domain (n = 2, *t*-test, alpha level 5%, two-tailed P = 0.26). This indicates that the N-domain does not need to be cleaved off for enzyme activation (Fig. 5b). Unexpectedly, the truncated LytU(70–192), containing the conserved M23 family domain was inactive but still structured, as inferred from its ^1^H, ^15^N HSQC spectrum (Supplementary Fig. S4). This underscores the importance of N-terminal residues in the catalytic domain for proper construction of the active site. In the LytU(49–192) structure (see below) residues I60-T63 form a strand-like conformation at the catalytic site end of the substrate-binding groove and they are thereby likely to assist in correct positioning of the catalytic residues. Moreover, Y67, located in the loop between the I60-T63 strand-like structure and β1 (residues 79–81), might have a role in catalysis as the corresponding Y204 in LytM was shown to be crucial for lytic activity^13^.

Both LytU forms, 151 Ile and Lys, are active, the lysine form being about 40% more efficient under the conditions studied. The activity significantly decreases at temperatures lower than 37 °C and increases when pH is raised from six to eight (Supplementary Fig. S5). The activity, particularly that of the Ile form, closely resembles that of active LytM(185–316) as could be expected considering the homology of the active sites. Most surprisingly, however, the deletion of the supposedly redundant LytU residue 151 rendered the enzyme completely inactive. The inactivation is not caused by a collapse of the protein structure, as the ^1^H, ^15^N HSQC spectrum indicates a folded protein (Supplementary Fig. S4). Of practical significance was a sharply increased activity when cofactors were other metal ions, namely Cu^2+^ and Co^2+^ (Fig. 5c). Pilot experiments exhibited further analogies with LytM^13^, Mn^2+^ reactivating the enzyme and Mg^2+^ and Ca^2+^ not having this effect, yet properties and diversity of potentially alternative cofactors remain a topic for future investigations. The differences in metal ion effects between pentaglycine cleavage activity measurements and cell lysis experiments (Supplementary Fig. S2) can be explained by the very different experimental setups. In NMR we observed the catalytic reaction between two pure components while in the autolysis assay the substrate is a whole cell. The CW is an exquisitely complex milieu with ion buffering capability and the measured outcome is the combined effect of a treatment on the substrate cell, i.e. CW components and all the autolytic activities present in the cells.

### LytU catalytic domain binds a second zinc that inhibits the enzyme

We observed a substantial reduction in the pentaglycine cleavage in response to the increasing concentration of zinc ions (Fig. 5b) and sought to understand underlying reasons for reduced activity. To this end, we measured ^1^H, ^15^N HSQC spectra under varying ion concentrations (Supplementary Figs S4 and S6). Indeed, ^1^H, ^15^N HSQC and histidine-optimized ^1^H, ^15^N HMBC spectra unequivocally showed that in the presence of a 2.5-fold excess of zinc ions the enzyme retains its fold and the catalytic H125 and H157 coordinate a second zinc ion. Although ^1^H, ^15^N HSQC spectra displayed a continuation of zinc ion binding also to LytM(185–316) (Supplementary Fig. S4), even a 5-fold molar excess of ion reduced catalytic activity by a mere 16%. Since no structure of a LytM domain with a second bound Zn^2+^ has been reported and inhibition has not previously been demonstrated, we conclude that this property is unique to LytU. Notably, a partial cleavage activity was observed for the apo LytU is likely due to tight metal binding of the protein and regaining of a limited activity by acquiring ions accessible as unavoidable trace impurities in the preceding concentration and reaction incubation steps.

Owing to the observed slow dissociation of the LytU–metal ion complexes in the NMR spectra, we resorted to isothermal titration calorimetry (ITC) to glean the information on zinc ion binding affinity and thermodynamics (Supplementary Fig. S7 and Table S2). The ITC data indicated that binding affinity for the first site is very high: K_d_ values are 0.26 and 0.22 nM for the Ile and Lys forms of LytU, respectively. These values are yet higher than the K_d_ = 18.5 nM determined for the LytM domain of *N. meningitidis* protein NMB0315^26^. Binding of the second zinc, although weaker by three orders of magnitude, is still submicromolar (0.32 and 0.49 µM for LytU Ile and Lys forms, respectively) and thus physiologically relevant.

We sought to identify additional side chains that would coordinate the second zinc ion in LytU. Of the available spatially proximal polar residues, N148 side chain showed large chemical shift perturbations (CSPs) in the ^1^H, ^15^N HSQC spectrum upon uptake of the second zinc ion. The residue Q152 side chain could also reach the histidine-coordinated ion. However, the N148A or N148S/Q152A mutations did not abolish binding of the second zinc, effectively eliminating them as candidates. Curiously, pentaglycine cleavage experiments revealed that N148A lost its activity. Its ^1^H, ^15^N HSQC showed a perfectly well-folded protein (Supplementary Fig. S4) with CSPs located in the neighbourhood of the asparagine when compared to the wild-type LytU, thus excluding the lack of proper folding as the cause of loss of activity.

### Binding of the second zinc is pH-dependent

**Figure 6:**
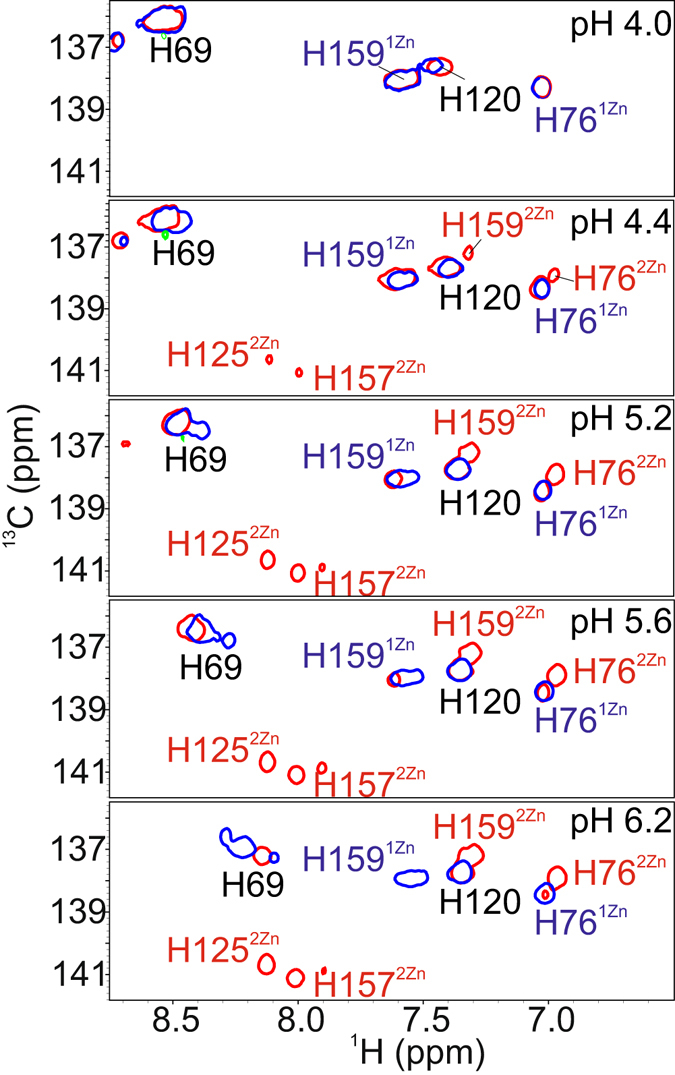
pH dependence of the second zinc binding. ^1^H, ^13^C HSQC spectra encompassing the aromatic peak region were acquired for one- and two-zinc LytU with ~0.4 pH unit intervals. Only titration figures at the lower end of the range (4.0–6.2) are provided due to the lack of differences at higher pH values. Shown are the overlays of the histidine ε1 region of one- and two-zinc LytU spectra at different pHs. One-zinc LytU is represented with blue contours and two-zinc LytU with red contours. At low pH, the C^ε1^-H^ε1^ regions of the ^1^H, ^13^C HSQC spectra of one- and two-zinc LytU are identical: H76 and H159 ε1 peaks have same positions and no peaks are observed for H125 or H157. When pH is raised, H76 and H159 ε1 peaks at positions corresponding to the two-zinc form appear and gain intensity. In addition, H125 and H157 ε1 peaks emerge. At pH 5.2 the one- and two-zinc His ε1 peaks have approximately equal intensity, and at pH over 6.5 one-zinc LytU disappears from the two-zinc LytU sample.

We followed the behaviour of histidine δ2, ε1 peaks in a series of ^1^H, ^13^C HSQC spectra acquired from a two-zinc LytU sample as a function of pH. At low pH conditions the ^1^H, ^13^C HSQC spectrum is identical to that acquired from a one-zinc LytU sample at same pH (Fig. 6). With the rising pH, signals corresponding to those of the two-zinc form start to appear, and by pH 5.2 peaks of both forms have an approximately equal intensity, until at pH over 6.5 peaks corresponding to the one-zinc form disappear from the two-zinc sample. This indicates that at pH 6.5 and above, if zinc ions are present, the catalytic histidines exist in an inhibited two-zinc form. The ^1^H, ^13^C HSQC spectra of apo and one-zinc LytU drastically differ for the whole studied pH range (4.0–8.2), indicating that binding of the first zinc ion to LytU is pH-independent.

### LytU structures

**Figure 7:**
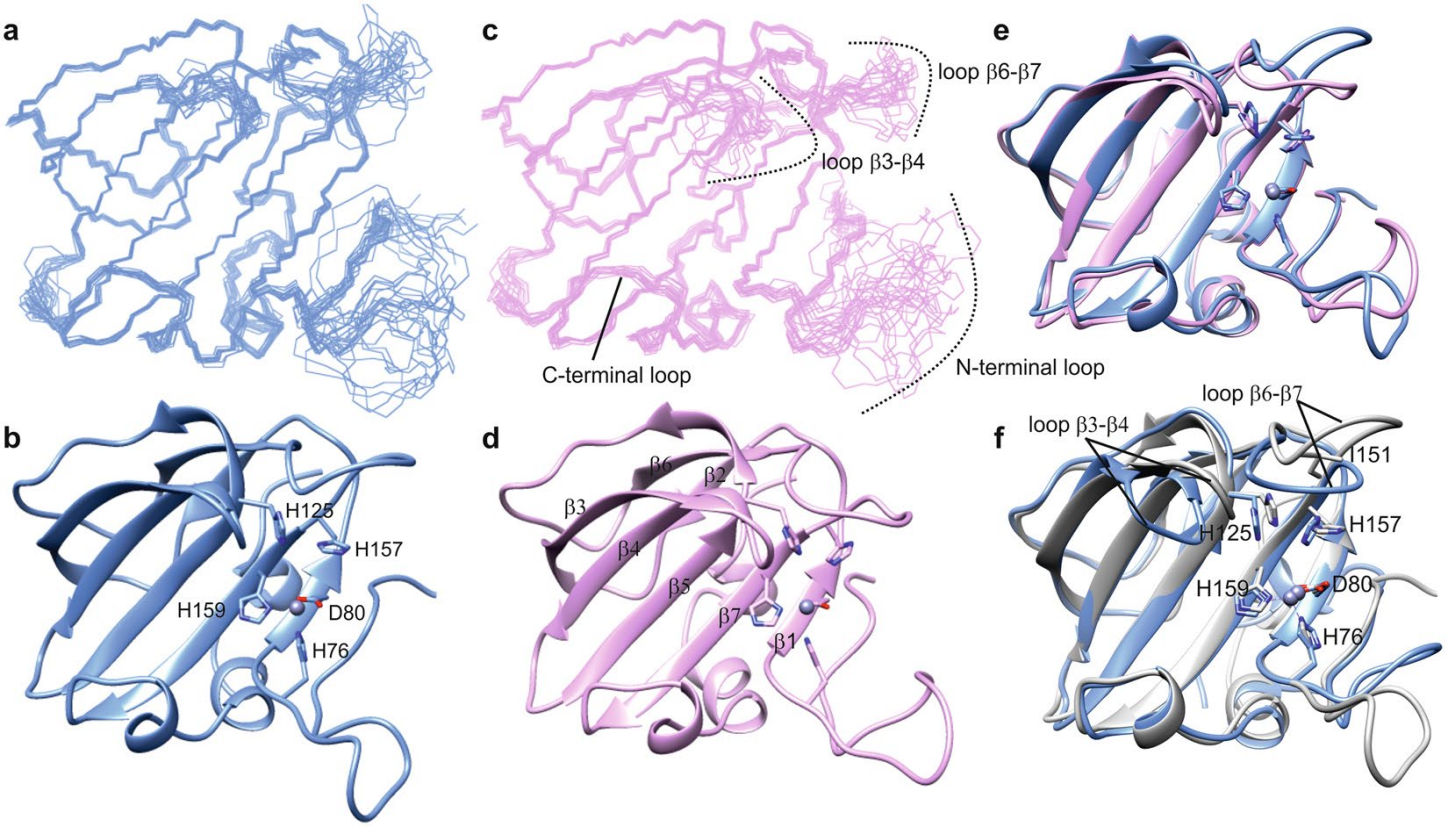
Structure of LytU and superposition to LytM catalytic domain. **(a** and **c)** Ensembles of fifteen structures of the least restraint violations of one- and two-zinc LytU, respectively. Backbone and heavy atom RMSDs for ordered residues of one- and two-zinc LytU are 0.3 and 0.7 Å, respectively. Residues shown are 57-192. **(b** and **d)** The lowest-energy structures represented in ribbon diagrams, for one- and two-zinc LytU, respectively. The substrate-binding groove can be seen horizontally, with the zinc-binding residues on the right. It is composed of four strands: β1:79-82, β3:95-103, β4:107-114, β5:119-129, and β7:158-164 and four loops: N-terminal loop: 64-78, loops β2-β3:104-106 and β6-β7:147-157, and C-terminal loop: 165-171, the latter of which includes a 3_10_ helical stretch. **(e)** Superposition of one- (blue) and two-zinc (pink) LytU. (**f**) Superposition of 1Zn LytU (blue) and LytM (PDB ID 2B13, grey). LytU is strikingly similar to LytM with an RMSD over backbone atoms in secondary structures of 0.9 Å. Residues shown are the zinc-coordinating H76, D80, H159 as well as the catalytic H125 and H157. The zinc ion is shown as a grey sphere. The position of the dimorphic residue of LytU, I151 in this case, is indicated in the β6-β7 loop. The structure of two-zinc LytU was determined with restraints for the first, tighter binding zinc only, and therefore the second zinc is not present in panels 2d and 2e. The figure was generated with the program UCSF Chimera^48^.

LytU shares the overall fold found in other lysostaphin peptidases, in which the prominent common feature is the groove formed by five β-strands flanked by four loops (Fig. 7 and Supplementary Table S2). The one- and two-zinc LytU structures can be superimposed with an RMSD over backbone atoms in β-strands of 0.4 Å (Fig. 7e). However, in the two-zinc LytU form the average local displacement of backbone atoms is significantly smaller in loop β6-β7, adjacent to the catalytic histidines, as well as in the side chain heavy atoms of H125 and H157. This is likely to be the outcome of structural rigidity entailed by zinc ion coordination to H125 and H157 as well as ensuing outer sphere effects. The latter include the appearance of S146 and T149 hydroxyl NOEs in the two-zinc LytU, resulting from stabilization of these protons through e.g. hydrogen bonding. Interestingly, ^15^N relaxation data (Supplementary Fig. S8) show that the region encompassing residues 64–75 in the N-terminal loop becomes more rigid upon binding the second zinc ion. This might originate from residue Y67 taking part in secondary zinc ion binding interactions. A catalytic role has been suggested for the corresponding tyrosine in LytM^13^.

LytU is strikingly similar to LytM (Fig. 7f). The immediate surroundings of the catalytic histidines are, however, dissimilar, which lays bases for the different affinities for the second zinc. Due to the likely conformational restrictions induced by P290 as well as the presence of several polar residues in the β6-β7 loop in LytM, the catalytic histidines in LytM reside in a more constricted and polar environment than those in LytU. Most importantly, in LytM serine S287 resides next to the catalytic histidines whereas in LytU the unique, hydrophobic residue I151 is packed between them. These distinct interactions with the aromatic rings are likely to influence histidine pK_a_s and their ability to coordinate zinc ions.

## Discussion

In comparison with other lysostaphin-related endopeptidases, LytU is relatively short, consisting only of an N-terminal membrane anchor, a linker region, and an endopeptidase domain. We showed that the enzyme is bound to the plasma membrane and that the endopeptidase domain resides outwards the membrane. Deletion and overexpression experiments both indicated that LytU has a direct role in the opening of the CW PG network for separation of daughter cells during cell division. The situation is quite intriguing as we have no evidence on specific cleavage and release of the LytU endopeptidase domain into the CW, and Matias and Beveridge have shown by cryo-EM that a periplasmic space exists in *S. aureus*, the thickness of which is about 16 nm in the cell envelope as well as in the septum^27^. Consequently, if the linker region is in a fully extended form the membrane-bound endopeptidase domain of LytU can reach about ~2/3 of the distance between the membrane and PG. But is the release of the endopeptidase domain needed *in vivo*? Considering, that the cell is a continuously moving, flexible structure it is most likely that occasionally the periplasmic space expands and shrinks, enabling LytU to take part in PG remodelling. Moreover, the tearing edge structure of the LytU mutant seems to be limited to the outermost surface layer of the CW only, which would be consistent with LytU cutting the PG network from the inside at the cell division site. Furthermore, most likely it is neither necessary nor desirable to cut PG completely as the LytU-cleaved PG lattice should be able to reform and stretch with the expansion, and eventually to smooth out into the newly synthesised CW. Regulated limited cleavage of PG pentaglycine bridges from the inside would ensure that the CW is never completely opened.

**Figure 8:**
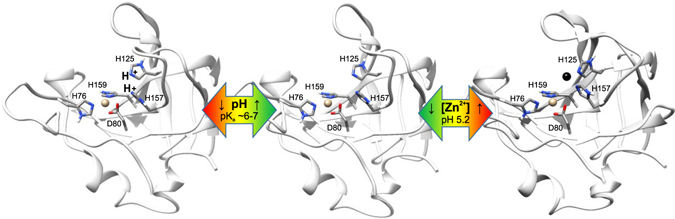
The constraints of LytU catalysis by inactivation with pH and zinc ions. Three structures of LytU catalytic domain are presented to the viewer peering through substrate binding groove (upper left in all three structures) with the site of catalysis at the proximal end. The key conserved residues are indicated. H76, D80 and H159 coordinate zinc (tan sphere) mandatory for substrate cleavage. Catalytic H157 and H125 reside nearby and are subjects to one of three fates. The block arrows indicate the driving forces in respective transitions and their gradient colour emphasizes positive (green) or negative (red) effect on LytU catalytic potential. The enzyme is in an active form (centre) and rapidly loses its activity (left) with decreasing pH (Supplementary Fig. S5). This is consistent with the protonation of H125 and H157. On the other hand, a second zinc ion (black sphere, right) may also bind LytU with a submicromolar dissociation constant (Supplementary Figs S6, S7 and Supplementary Table S2) and it is coordinated by N^ε2^ of the H125 and H157 (Supplementary Fig. S6). Consequently, this binding is catalytically inhibitory. Supplementary Fig. S10 provides a close-up look at the tautomeric forms of histidines.

Though LytU is not strictly needed in cell division, as shown by the deletion data and normal CW dimensions in EM pictures, its involvement cannot be excluded. This calls for attention to the regulation of LytU activity. Particularly so, as we demonstrated that purified LytU is an effective anti-bacterial agent that lyses *S. aureus* cells, the very same cells it originates from. Although other related autolytic enzymes, e.g. LytM and NMB0315, possess large auto-inhibitory domains^20, 26^, the 26–48 residue region upstream of the catalytic domain in LytU has no effect on catalysis *in vitro* and therefore it does not serve in any apparent regulatory function. Naturally, it is possible that this region serves in other functions, like protein–protein or protein–CW component interactions, or simply acts as an entropic spring that may direct the role of the protein. Our *in vitro* NMR spectroscopic structural and functional characterization of soluble LytU revealed two mechanisms that can explain how the enzyme is regulated: LytU catalytic activity is sensitive to pH and metal ion content. A simple molecular model (Fig. 8, Supplementary Fig. 10) illustrates how acidity and metal ion availability controls the catalytic activity of LytU. At low pH, protons incapacitate catalytic H125 and 157, whereas, at higher pH, an excess of Zn^2+^ acts the same.

The pH-sensitivity of CW autolysins is a well-known phenomenon^28^. Its cellular mechanism was recently elucidated for *S. aureus* AtlA, whose pH-dependent control of autolysis activity was demonstrated to be the outcome of an intimate interaction between membrane proton gradient and wall teichoic acids, WTAs^29^. WTAs, which are bound to PG of CW, and lipoteichoic acids (LTAs), which are tethered to the cell membrane but extend through the periplasmic space into PG as well, are zwitterionic CW polymers. Together they are referred to as teichoic acids (TAs). Due to their extensive phosphate group network, TAs contribute to the bulk of negative charge in the CW, which is counterbalanced by protons. Together they orchestrate the pH in CW and activity of AltA. The ability of itinerant metal ions to compete for the negative charges of TAs adds a new dimension to the observed LytU regulation.

Blood concentrations of zinc are in 9.2–20 µM range and the ion is trafficked by anionic phosphate in CW. To date, the most comprehensive and consolidating model of metal ion binding was proposed by Thomas and Rice in isolated *B. subtilis* CW^30^. It was described as an electrostatic phenomenon at low ionic strengths and two zones of binding were demonstrated: strong initial binding followed by a negative cooperativity-induced weaker binding. These can depend on the environment of the cell. The overall K_d_ values for Ca^2+^ and Mg^2+^ at pH 5.65 were 1.43 and 1.49 µM, respectively. Taking into account similar values determined for Zn^2+^, Ca^2+^, and Mg^2+^ in unrelated studies, as well as our data for the first and second zinc binding sites (K_d_ values 0.2–0.3 nM and 0.32–0.49 µM, respectively), it can be concluded that both LytU sites can successfully compete with CW for metal ion binding.

We observed that overproduction of LytU-Strep completely blocks daughter cell separation and that LytU is found exclusively in the plasma membrane where it has an even distribution. This calls for specific localisation or activation of LytU at the site of septal assembly. It is quite likely that assembly of all the components involved in the formation of the septa for cell division, and subsequent synthesis of the new plasma membrane excludes, for example, electron transfer and ATP-synthesis complexes from the area from which the CW will be cleaved. This alone could lead to changes in local pH (proton gradient) and subsequently in concentrations of divalent cations (either transported or already present in the CW). It could also locally alter the distance between the membrane-bound LytU and PG substrate during the CW opening. This would be in accordance with overproduced C-terminally Strep-tagged LytU completely and specifically distorting the assembly and abolishing cutting of CW PG at the cell division sites.

The inhibitory effect of a second zinc in a dinuclear zinc site has been described for several zinc proteases, such as carboxypeptidase A^31^, thermolysin^32^ and subclass B2 metallo-β-lactamases^33^, but to our knowledge, LytU is to date the only member of the M23 glycylglycine endopeptidase family shown to bind two zinc ions, and thereby potentially influence its activity.

Alluring similarities were found between LytU and CphA, a B2 β-lactamase from *Aeromonas hydrophila*. Despite their different overall structures and substrates (CphA has an αββα fold typical for metallo-β-lactamases, in which a ββ sandwich is surrounded by helices^34^), the two proteins share some key features. First, their zinc-coordinating atoms structurally align nicely (Supplementary Fig. S9) although the proteins differ by one zinc-coordinating residue: H159 of LytU corresponds to C221 in CphA. In CphA a water molecule participates in the coordination of the second inhibitory zinc. Second, the K_d1_ and K_d2_ values of zinc ion binding are in a similar range, <20 nM and 46 μM, and 0.2 nM and 0.3 μM for CphA and LytU, respectively. And third, in both proteins binding of the second Zn is pH-dependent, with increasing inhibition at higher pH^33^. Considering our fruitless mutational experiments of polar side chains to find additional zinc-coordinating residues in LytU and the structural and functional similarities between LytU and CphA we presume that water can act as a ligand for the zinc ion also in LytU. This water molecule is not likely to be stable in LytU because the nearby protons showed no pertinent NOE signals. The fourth ligand in CphA is a sulfate ion from the crystallization solution. Our NMR sample contained solely ZnCl_2_. In principle, also chloride could occupy the vacant coordination position(s). This type of coordination has been observed in Carboxypeptidase A^31^. In this enzyme, a distorted tetrahedral coordination, formed by one glutamate carboxylate oxygen, two water molecules and a chloride ion, is observed for the second zinc ion.

The binding of zinc ion to the catalytic histidines H118 and H196 in CphA prevents them from exercising their roles in the catalysis. Specifically, H118 acts as a general base to activate a water molecule and H196 polarizes the carbonyl oxygen of the β-lactam bond by forming a hydrogen bond^34^. The very recent structure of LytM in complex with a substrate analogue^13^ revealed details on the M23 lysostaphin peptidase family catalytic mechanism. Analogously to CphA, LytM H260 and H291 are essential for catalysis. The first histidine abstracts a proton from a water molecule generating a nucleophilic hydroxide ion, which subsequently attacks the carbonyl carbon of the peptide bond. The second histidine stabilizes the tetrahedral transition state. The inhibitory zinc ion in LytU acts in a manner similar to that proposed for CphA. LytU ^1^H, ^15^N HMBC data clearly indicates that the corresponding histidines H125 and H157 coordinate the zinc ion in the 2Zn form which renders them incapable of performing their roles in catalysis.

The participation of Y204 in the catalytic mechanism of LytM has been exposed^13^. Its hydroxyl group can stabilize the tetrahedral transition state. This residue corresponds to LytU Y67, which is structurally oriented like Y204 in LytM, pointing towards the catalytic zinc ion, thereby suggesting a similar role in pentaglycine cleavage for Y67 as well. This is further supported by LytU(70–192) being catalytically inactive yet preserving its structural integrity.

LytU catalytic domain exhibits canonical features of lysostaphin/LytM kin and secondary structures of LytU and its closest homologue LytM are faithfully superimposable with very similar orientations of active site residues. Nonetheless, the role of loop β6-β7 in catalytic activity has not been previously reported. Interestingly, mutation N148A abolished enzyme activity completely although it did not have any influence on the structural integrity of LytU nor binding of second zinc. Most surprisingly, the residue 151, which is unique among the family, is obligatory for the enzyme to be active. Our attempts to produce a LytM protein with the equivalent Ile and Lys insertions were unsuccessful, as it appeared that the mutations made LytM insoluble.

## Materials and Methods

### Generation of LytU deletion mutant

The sa0205::intron mutation (LytU deficient mutant strain) was constructed into the *S. aureus* Newman strain using TargeTron system (Sigma-Aldrich) as previously described^35^. Mutating L1.LtrB intron insertion was made at position 237/238 in the sa0205 gene using primers IBS, EBSS1d, and EBS2 with the specific sequences listed in Supplementary Table S1. The resulting new strain RH7796 was verified by sequencing.

### Generation of LytU overexpression construct

For controlled expression and detection of LytU expression in cells a plasmid shuttle vector was modified as previously described^35^. Complete sa0205 gene with an added C-terminal Strep-tag II tail was cloned between the EcoRI and BamHI sites in pEPSA5 vector (primers SA0205 pEP fw and SA0205 pEP rv, Supplementary Table S1). The resulting plasmid pKTH3813 was verified by sequencing and then transferred from E. coli DH5α first to *S. aureus* RN4220 and then into the Newman strain to yield strains RH7777 and RH7781, respectively.

### Construction of GFP-LytU fusion protein

pWKD56f E. coli-*S. aureus* shuttle plasmid^36^ was modified for expression of GFPuv4-LytU in *S. aureus* and determination of LytU localization pattern in the cell as follows. The NotI-PstI fragment of pWKD56f containing the Pxyl-gfpuv4 construct was PCR-amplified with the Pxylgfp4 primers (Supplementary Table S1) and inserted into pWKD56f cut with NotI and PstI. This replacement was performed to insert AvrII and PacI restriction sites after the coding sequence of GFPuv4. The obtained plasmid pJUNK1 was then cut with AvrII and PacI to insert the PCR amplified coding sequence of LytU to which the same restriction sites were added with primers SA0205pMK4 (Supplementary Table S1) in frame with gfpuv4. The obtained plasmid pJUNK2 was then cut with PacI and PstI to add a synthetic transcription terminator t1R between the restriction sites (t1R primers, Supplementary Table S1) to yield plasmid pJUNK3. This plasmid was transformed into *S. aureus* RN4220 with selection for chloramphenicol resistance (10 µg/ml). pJUNK3 is a derivative of pMK4 (the accession number of pMK4 in GenBank is EU549779) with the replicon of pC194 having the copy number of 15 per cell.

### Production of purified recombinant proteins

For purification of different forms of recombinant proteins, chromosomal DNA from *S. aureus* Newman strain was used as the original PCR template. Primers used in this study have been listed in Supplementary Table S1 and the expression vector used was pGEX-2T to which inserts were cloned between the BamH1 and EcoR1 cloning sites. Constructs were confirmed by sequencing. Point mutants were obtained using *QuikChange II* site-directed mutagenesis kit (Agilent Technologies, USA) with the pGEX-2T carrying the previously cloned wild-type fragment as a template and results were confirmed by sequencing. For protein expression, all plasmids were transformed into *E. coli* BL21 (DE3) cells. Purification was carried out utilizing GST-tag and followed by subsequent tag cleavage with thrombin and removal in size exclusion chromatography. Detailed procedures for expression and purification of the proteins for structural and catalytic activity studies are provided in Supplementary Information.

### Fractionation

For localising LytU in cellular fractions overexpression strain RH7781 with and without 1% xylose induction was used. Cells grown in BHI medium were harvested with low-speed centrifugation, resuspended in 20 mM Tris pH 7.5 buffer, and ruptured using French Press. Lysate was cleared of cell debris by low-speed centrifugation (13 000 g, 4 °C) in 2 ml Eppendorf tubes and a second centrifugation of the supernatant was carried out in Beckman ultracentrifuge, 4 °C, 20 000 rpm, sw 41 rotor for 2 h to create the membrane containing pellet which was then dissolved in buffer. Samples of all fractions were analyzed in SDS-polyacryl amide mini gels and gels were stained with Coomassie blue using standard methods. When whole cells of *S.aureus* strains were analyzed in protein gels a brief treatment with lysostaphin (Sigma-Aldrich, USA) was used prior to sample preparation.

### Immunolocalization

*S. aureus* RH7781 was grown in BHI medium and 0.02% xylose was added at the cell density of Klett 70 to induce LytU-Strep expression for 1 hour after which cultures were harvested. Cells from 1 ml of culture were suspended in 0.1 ml of the protoplast buffer (20 mM potassium phosphate pH 7.5, 15 mM MgCl_2_ and 20% sucrose) containing 0.1 mg/ml lysostaphin for 40 min at 37 °C. Adequate success of protoplast conversion (clear majority) was verified with microscopic examination and cells were then harvested by centrifugation at 5 000 rpm in an Eppendorf miniSpin Plus centrifuge for 5 min. The protoplast pellet was resuspended in 0.1 ml of protoplast buffer containing 1 mg/ml trypsin and then incubated for 40 min at 37 °C. A parallel sample was incubated in a similar manner but without trypsin. After the trypsin treatment trypsin inhibitor was added to the final concentration of 1.2 mg/ml. The protoplasts and protoplast supernatant were heated in Laemmli sample buffer for 10 min at 100 °C and samples were then analysed for LytU-Strep, PrsA and TrxA levels with immunoblotting using anti-Strep-tag, anti-PrsA and anti-TrxA antibodies, respectively.

### Microscopy

Light and electron microscopy were carried out as previously described^35^. For light microscopy samples were Gram-stained. Confocal microscopy for LytU spatial localization in the membrane was carried out as follows. *S. aureus* RN4220 carrying pJUNK3 was cultivated in BHI medium in the presence of chloramphenicol (10 µg/ml) and 0.5% xylose was added at the cell density of OD_600_ 0.6 to induce GFPuv4-LytU. Samples were prepared for microscopy after 2 h induction. Cells from 1 ml of culture were harvested by centrifugation, washed once with 1 ml of PBS and resuspended in the same volume of PBS. A small drop of the cell suspension (2 µl) was spotted on a microscope slide, coved with a cover slip, and sealed with nail polish. The microscopy of the sample and non-induced control was performed with a Leica TCS-SP5 confocal laser-scanning microscope with excitation at 488 nm.

### Cell lysis experiments

The activity of purified LytU fragments against whole *S. aureus* cells was carried out using a Bioscreen C apparatus (Growth Curves, Finland) and autolysis experiment procedure as previously described^35^. Substrate cells were in this study grown either to logarithmic growth phase (3 h) or late stationary phase (fresh overnight cultures) prior to harvesting and substrate sample preparation. Samples were prepared in 20 mM Tris-HCl pH 7.0 buffer. Final concentrations of cations or EDTA were 0.05 mM and the final concentration of exogenous LytU, except for controls, was 4 μM.

### Enzyme activity measurement

Pentaglycine cleavage by recombinant proteins was carried out in multiple independent series of incubation samples and normalized to the activity of LytU-Ile with one Zn^2+^ ion. For protein comparison, incubations included 0.03–0.04 mM purified protein and 1–2 mM pentaglycine (Sigma-Aldrich) in PBS buffer. ZnCl_2_, CuCl_2_ and CoCl_2_ solutions were added as respective sources of metal ions to the desired protein-ion ratio. All incubations had 90% v/v D_2_O as a solvent for water signal suppression, and their total volume was 300 μl. Before the NMR analysis, reactions were incubated, in their linear range, for up to 70 hours and quenched by heating the samples for 15 min at 85 °C.

All incubations were carried out at 37 °C except when different temperatures were tested. Sodium phosphate buffers (150 mM) were used to test the effect of different pH. Before NMR analysis 0.5 mM trimethylsilyl propanoic acid (TSP) was added as a standard. Reaction progress and product formation were observed by recording a ^1^H spectrum at 37 °C with a Varian INOVA 800 MHz spectrometer manually or with a Bruker AVANCE 600 MHz spectrometer equipped with an autosampler. Data were processed with Bruker TopSpin 3.5 and integrated with Amix 3.9.12.

Assignment of pentaglycine and its cleavage products was performed with a ^1^H, ^13^C HMBC experiment in PBS buffer having 99% v/v D_2_O as a solvent. These data were acquired at 37 °C on a Bruker AVANCE III HD 800 MHz spectrometer, equipped with a TCI ^1^H/^13^C/^15^N cryoprobe. The stability of the substrate pentaglycine over the time span of the activity assay was studied with a control experiment presented in Supplementary Fig. S3.

### Isothermal titration calorimetry

ITC experiments were carried out at 35 °C using a MicroCal VP-ITC microcalorimeter. Purified catalytic domains of LytU were dialyzed extensively against cacodylate (**Note**: Extremely hazardous substance!) buffer (50 mM cacodylic acid, 100 mM NaCl, pH 7.4) to remove phosphate ions of the original purification buffer and subsequently concentrated. Titrations were carried out with 50 μM protein in the calorimeter cell and syringe contained 800 μM ZnCl_2_ dissolved in the final cacodylate dialysis buffer. Typical injection volume was 7 μl, a spacing between injections was 300 s, and stirring speed was 300 rpm. Baseline was obtained by titrating zinc chloride into the dialysate without protein and was subtracted from the sample titration experiments. The protein-zinc binding isotherms were generated by the nonlinear least-square fitting method of the data and employing a two-site model with MicroCal Origin 7 software. Thermodynamic parameters were obtained as mean values of at least two independent measurements.

### Protein NMR spectroscopy

NMR samples had a protein concentration of 0.5 mM in 20 mM Bis-Tris pH 6.5 buffer, and contained 7% D_2_O, except for histidine-optimized ^1^H, ^15^N HMBC spectra, where water was fully substituted with D_2_O. The tautomeric state of histidines and the Zn-coordination mode were derived from the cross peak patterns^37^ observed in histidine-optimized ^1^H, ^15^N HMBC spectra acquired from the 0, 1Zn and 2Zn forms of LytU. For zinc-coordinating histidines, this analysis was in accord with the coordination mode determined from the C^δ1^, C^ε1^ chemical shift difference^38^.

LytU(49–192) chemical shift assignment has been described previously^39^. NOESY spectra for structure determination were acquired at 35 °C on a Varian INOVA 800 MHz spectrometer equipped with a cryogenic probe head. Distance restraints were derived from 3D ^1^H, ^15^N NOESY-HSQC, ^1^H, ^13^C NOESY-HSQC and histidine nitrogen frequency-optimized 2D ^1^H, ^15^N NOESY-HSQC spectra. Structures were determined by the automated procedure in Cyana 2.1^40^ with the KEEP option for four manually assigned NOE peaks in both the 1Zn and 2Zn calculations. These assignments restrain distances between the first and the second β strands. We included one zinc in both structure calculations, with four restraints: the distance of H76 N^ε2^, D80 O^δ1^ and H159 N^δ1^ to Zn was restrained to 2.0–2.2 Å, and H76/H159 C^γ^ to Zn to 4.1–4.3 and 3.0–3.2 Å, respectively. Water is likely to complete the coordination sphere, but we found no evidence for a stable water molecule in the NOE spectra. Hence, no restraints for a fourth ligand were used. Out of 300 structures generated with Cyana, fifteen with the lowest target function were selected for subsequent AMBER 14^41^ minimization in explicit solvent. The atomic coordinates and structural restraints for 1Zn and 2Zn protein structures have been deposited in the Protein Data Bank (PDB) with the accession codes 5KQB and 5KQC.

### Data Availability

The accession codes for atomic coordinates and structural restraints of 1Zn and 2Zn protein structures in the Protein Data Bank (PDB) are 5KQB and 5KQC, respectively.

## Electronic supplementary material

Supplementary information
