# Supplementary material for: Identification and structural characterization of LytU, a unique peptidoglycan endopeptidase from the lysostaphin family
Source: Sci Rep. 2017 Jul 20;7:6020. doi: 10.1038/s41598-017-06135-w (PMC5519744; doi:10.1038/s41598-017-06135-w)
Supplement: Supplementary file 1 — Supplementary information [file 41598_2017_6135_MOESM1_ESM.pdf]

Identification and structural characterization of LytU, a unique peptidoglycan endopeptidase from the lysostaphin family

Vytas Raulinaitis, Helena Tossavainen, Olli Aitio, Jarmo T. Juuti, Keiichi Hiramatsu, Vesa Kontinen and Perttu Permi*

*Corresponding author: Perttu Permi, Nanoscience Center, P.O. Box 35, FI-40014, University of Jyvaskyla, Finland. E-mail: perttu.permi@jyu.fi, tel. +358 40 8054288.

Supplementary Information

Supplementary Table S1. **Primers used in this study.** See material and methods for further details on use of the primers. The primers were obtained from Oligomer, Finland.

**Primers**	**Direction**	**5′-3′ Sequence**
LytU*wt*-26-5′	Forward	CGCGGATCCGATGACATTCAAAAATGGTTTAACC
LytU*wt*-33-5′	Forward	CGC GGA TCC AAC CAA TAT ACC GAT AAA TTG AC
LytU*wt*-47-5′	Forward	CGCGGATCCGGACACTCAAAATGGGAAGAC
LytU*wt*-49-5′	Forward	CGCGGATCCAAATGGGAAGACTTTTTAGAGGG
LytU*wt*-70-5′	Forward	CGCGGATCCTCA CCATTTGATGGTAAGCATTATG
LytU*wt*-192-3′	Reverse	CGCGAATTCTTACTACAAATCATATAGGCTACGTTCCC
LytM*wt*-185-5′	Forward	CGCGGATCCCATGCGAAAGACGCAAG
LytM*wt*-316-3′	Reverse	CGCGAATTCTTATCTACTTTGCAAGTATGACGTTG
LytU-Δ151	Sense	TTCAGGCAATACAGGTCAAACGACAGGCGCAC
	Antisense	GTGCGCCTGTCGTTTGACCTGTATTGCCTGAA
LytU-I151K	Sense	TGCATATTCAGGCAATACAGGTAAACAAACGACAGGCG
	Antisense	ACGTATAAGTCCGTTATGTCCATTTGTTTGCTGTCCGC
LytU-N148A	Sense	GATATTATTGCATATTCAGGCGCTACAGGTATACAAACGACAGG
	Antisense	CCTGTCGTTTGTATACCTGTAGCGCCTGAATATGCAATAATATC
LytU-N148S/Q152A	Sense	GCATATTCAGGCAGTACAGGTATAGCAACGACAGGCGCAC
	Antisense	GTGCGCCTGTCGTTGCTATACCTGTACTGCCTGAATATGC
LytM-286I	Sense	CAGGTAGTACGGGTATTAATTCAACAGCGCCTC
	Antisense	GAGGCGCTGTTGAATTAATACCCGTACTACCTG
LytM-286K	Sense	CAGGTAGTACGGGTAAAAATTCAACAGCGCCTCAC
	Antisense	GTGAGGCGCTGTTGAATTTTTACCCGTACTACCTG
Pxylgfp	Forward	TCCCCCGGGGCGGCCGCGTTAAGA
	Reverse	AAAACTGCAGAATTAATTAAAAACCTAGGTTTGTAGAGCTCATCCATGCCA
SA0205pMK4	Forward	CACACCTAGGATGACAAAGCGACCAAAACGT
	Reverse	CACATTAATTAACTACAAATCATATAGGCTACGTTCCCC
t1R	Forward	TAAATGCATATGAGTGGCTAGCATTTTTCCACTCATCTGCA
	Reverse	GATGAGTGGAAAAATGCTAGCCACTCATATGCATTTAAT
IBS		AAAAAAGCTTATAATTATCCTTACATTACGGCATTGTGCGCCCAGATAGGGTG
EBS1d		CAGATTGTACAAATGTGGTGATAACAGATAAGTCGGCATTGATAACTTACCTTTCTTTGT
EBS2:		TGAACGCAAGTTTCTAATTTCGGTTTAATGTCGATAGAGGAAAGTGTCT
SA0205 pEP fw		CACAGAATTCAAAATATATGAAGGATGGATGCCACT
SA0205 pEP rv		CACAGGATCCCTACTTTTCGAACTGCGGGTGGCTCCACAAATCATATAGGCTACGTTCCCC

Supplementary Table S2. NMR constraints and structural statistics for the ensembles of the 15 lowest-energy structures of 1Zn and 2Zn LytU.

**Completeness of resonance assignments**[a]	**1Zn**	**2Zn**
Backbone (%)	92.5	89.6
Side chain, aliphatic (%)	97.6	95.4
aromatic (%)	83.6	95.7
**Experimental restraints**		
Distance restraints		
Total	2946	2873
Intraresidual (i=j)	592	586
Sequential (i-j=1)	673	646
Medium range (1<i-j<5)	380	374
Long range (i-j≥5)	1301	1267
No. of restraints per restrained residue	20.9	20.5
No. of long-range restraints per restrained residue	9.2	9.1
**Residual restraints violations**		
Average no. of distance violations per structure		
0.1-0.2 Å	0.13 (max 0.23 Å)	0.07
0.2-0.5 Å	0	0.20 (max 0.38 Å)
>0.5 Å	0	0
**Model quality**[b]		
RMSD backbone atoms (Å)	0.4	0.3
RMSD heavy atoms (Å)	0.8	0.7
RMSD bond lengths (Å)	0.014	0.014
RMSD bond angles (°)	2.2	2.2
**Molprobity Ramachandran statistics**[b]		
Most favoured regions (%)	95	96.7
Allowed regions (%)	5	3.3
Disallowed regions (%)	0	0
**Global quality scores (raw/Z score)**[b]		
Verify3D	0.42/-0.64	0.44/-0.32
ProsaII	0.22/-1.78	0.23/-1.74
PROCHECK(ϕ-ψ)	-0.54/-1.81	-0.46/-1.49
PROCHECK (all)	-0.48/-2.84	-0.43/-2.54
Molprobity clash score	1.75/1.23	0.86/1.38
**Model contents**		
Ordered residues	104	109
Total no. of residues	145	145
BMRB accession number	30130	30131
PDB ID code	5KQB	5KQC

[a]Backbone includes C^{α}, C^{β}, N and H atoms, except the N-terminal amide. For side chains, excluded are the highly exchangeable groups (Lys, amino, Arg, guanido, Ser/Thr/Tyr hydroxyl, $His^{\delta1/\delta2}$), as well as all non-protonated carbons and nitrogens. [b]Ordered residues: 77-103,108-148,157-192 for 1Zn and 60-64,78-104,107-148,158-192 for 2Zn forms.

Protein expression and purification

Constructed expression plasmids were transformed into *E. coli* BL21 (DE3) cells (Merck Biosciences, Germany) for production and the strains (logarithmic growth phase liquid cultures) were stored frozen in 15% v/v glycerol at -70 °C in aliquots.

Cultures were started from the storage vials by inoculating 50 ml of LB medium containing 0.1 mg/ml ampicillin for overnight (16 hr) at 30 °C in a shaker (220 rpm). The following day, this inoculum culture was diluted with 2 L of LB medium (0.1 mg/ml ampicillin) prior to dividing it into four 2 L flasks for continued growing at 37 °C shaker. Protein expression was induced by adding IPTG (Thermo Fisher Scientific) to 0.5 mM final concentration when optical density A_{600} reached 0.5 – 0.6. Cultures were then incubated for 3 h at 30 °C and harvested by centrifugation (2000 g, 4 °C, 15 min). Pellets were resuspended in 20 ml of PBS and either subjected to immediate protein purification or flash frozen in liquid nitrogen and stored at -70 °C.

If isotopically labelled (^{15}N, ^{13}C) proteins were needed, the overnight culture was centrifuged (2000 g, 4 °C, 15 min) and pellets were resuspended into 50 ml of PBS. Centrifugation was then repeated, to further remove unlabelled nutrients of LB medium. Pellets were then resuspended into 2 l of M9 minimal medium with 0.1 mg/ml ampicillin and culturing was continued as describe above. The sole labelled nitrogen and carbon sources in the M9 medium were 1 g/l $^{15}NH_4Cl$ and 2 g/l D-glucose-$^{13}C_6$ (Cambridge Isotope Laboratories, USA), respectively. After induction, these cultures were, however, incubated for 4 hr at 37 °C instead of lowering the incubation temperature.

Harvested cells were treated with 0.5 ml of 1 mg/ml DNAse (Roche Diagnostics, Switzerland) before cell lysis. Cells were lysed either with French Pressure Cell Disrupter (Thermo Fisher Scientific) or by using Branson Sonifier B15 cell disruptor (Emerson Electric, USA) The cell lysate was centrifuged for 1 h at 4000 g, 4 °C, and the resulting supernatant was filtered through a Millipore 0.2 µm polyethersulfone filter (Merck Biosciences). Filtrated lysate was loaded onto affinity chromatography in 3 ml Glutathione Sepharose 4 Fast Flow resin (GE Healthcare Life Sciences). The loading flow rate was 0.5 ml/min. The resin with bound protein was subsequently washed with 10 ml of PBS at 1 ml/min flow rate. Then 12 ml of PBS with 200 units of thrombin (GE Healthcare Life Sciences) was added to the column with the resin bound to GST-tagged protein and the column was sealed on both ends. Sealed column was then incubated for 16 hr at room temperature with gentle top over bottom rotation. The buffer with the released protein of interest was collected and concentrated using Millipore 3000 MWCO spin-concentrators (Merck Biosciences). The protein was further purified by gel filtration on HiLoad 16/60 Superdex 75 column (GE Healthcare Life Sciences) connected to Äktapurifier (GE Healthcare Life Sciences) using 1 ml/min PBS mobile phase (retention time was around 80 min). Fractions containing the protein were pooled (total of 10 to 12 ml) and protein concentration was measured using NanoDrop 2000c spectrophotometer (Thermo Fisher Scientific). A 100-fold molar excess of EDTA was added to remove any protein bound metal ions. After incubation with EDTA for 1 hr at room temperature, with gentle shaking, the protein solution was transferred to 6-8000 MWCO tubular dialysis membrane (Spectrum Laboratories, USA) and dialysed against 5 l of PBS at 4 °C. Dialysis buffer was changed after 2, 4, and 14 hours. Dialysed protein was then concentrated with Millipore 3000 MWCO spin-concentrators (Merck Biosciences) and in the process, when needed, the buffer was changed to 20 mM Bis-Tris, pH 6.5. The typical yields were about 10 mg of ^{15}N, ^{13}C-labelled protein and about 20 mg of unlabelled protein. Metal ion (Zn^{2+}) was added to the final preparation as needed. Proteins were stored in the buffer either at -20 °C or lyophilized for short and long term storage, respectively.

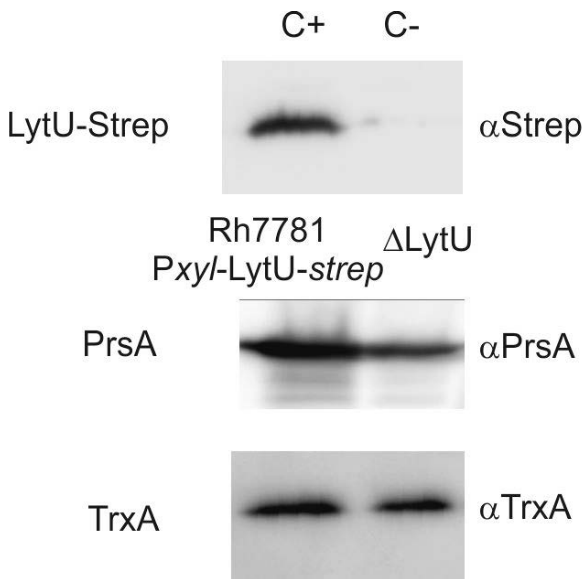

Supplementary Figure S1. Assay controls of the fractionation experiment. C+, positive control for immunoreactions with saturated LytU-Strep (strain RH7781, whole cells) sample without trypsin treatment; **C-**, LytU-deficient mutant strain RH7796 (whole cells) treated like the C+ control and serving as a negative control for LytU. As the level of production from the used expression vector is controlled by the amount of xylose inducer, 0.02 % xylose was used to get very low amounts of LytU, resembling the natural conditions, and 0.5 % to obtain a sample for LytU-saturated positive control of the antibody reactions. Antibodies: αStrep, a commercial antibody against the Strep II tag; αPrsA, a polyclonal antibody raised against a known surface protein on the outside of the plasma membrane and αStrxA, a polyclonal antibody raised against a cytoplasmic thioredoxin reductase complex component.

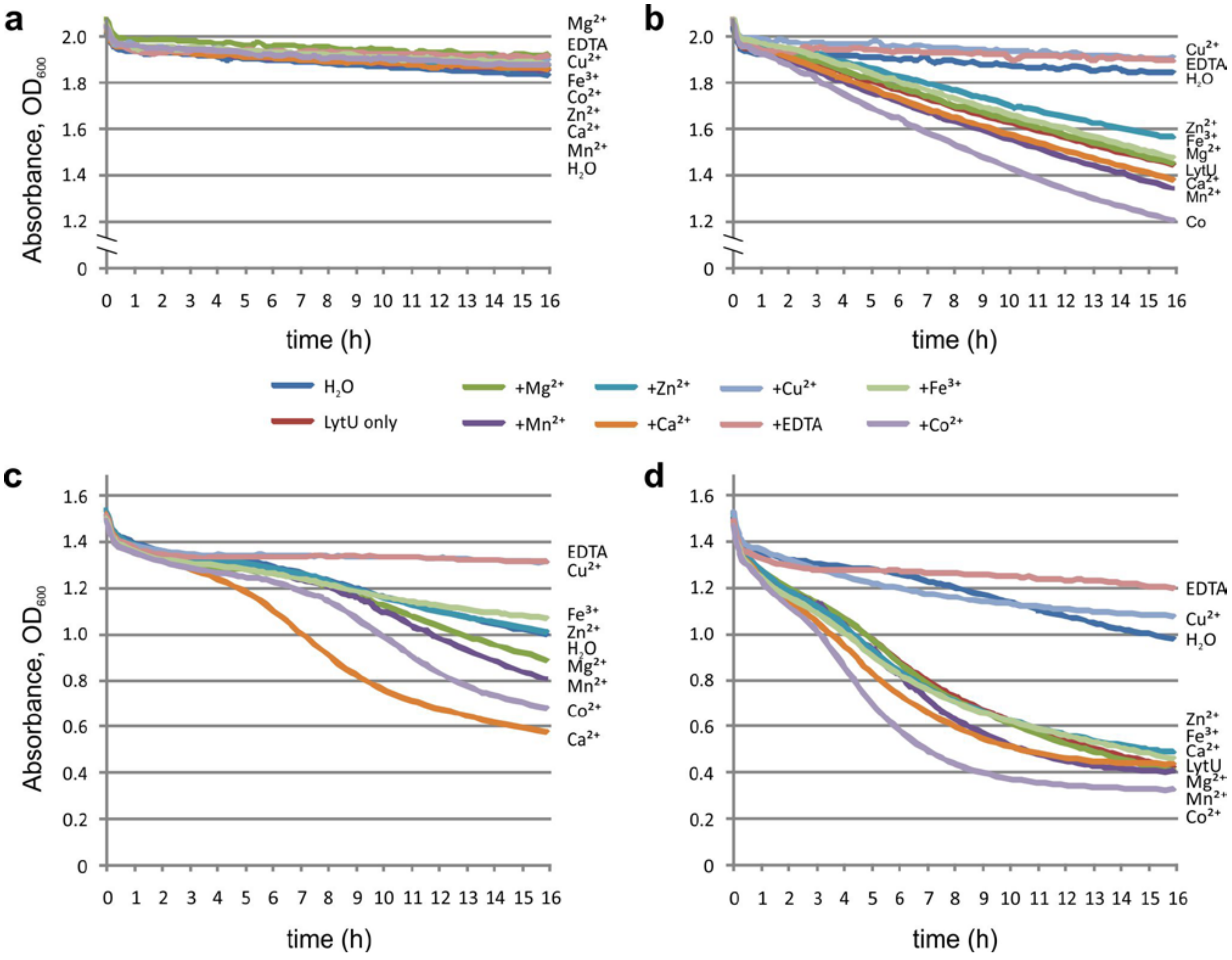

Supplementary Figure S2. Lytic activity of LytU(33-192) protein against *S. aureus* Newman cells. (a) Control experiment (i.e. pure autolysis experiment) with resting (late stationary) cells as a substrate without added purified protein. Effect of 0.05 mM (final) additions of different divalent cations (chloride salts), $FeCl_3$ (Fe^{2+} had a neutral and Fe^{3+} a slightly inhibitory effect on LytU activity in pretesting) and EDTA on autolysis of cells in 20 mM Tris-HCl pH 7 buffer are shown. **(b)** Same experiment as in panel a, but purified LytU has been added to 4 μM concentration (except in water only control). **(c)** Similar control experiment as in panel a, but substrate cells were harvested from an actively growing (3 h) bacterial culture. **(d)** Same experiment as in panel c, but purified LytU(33-192) was added as in panel b. Means of five replicates are shown for each line to maintain readability as replicates were practically overlapping.

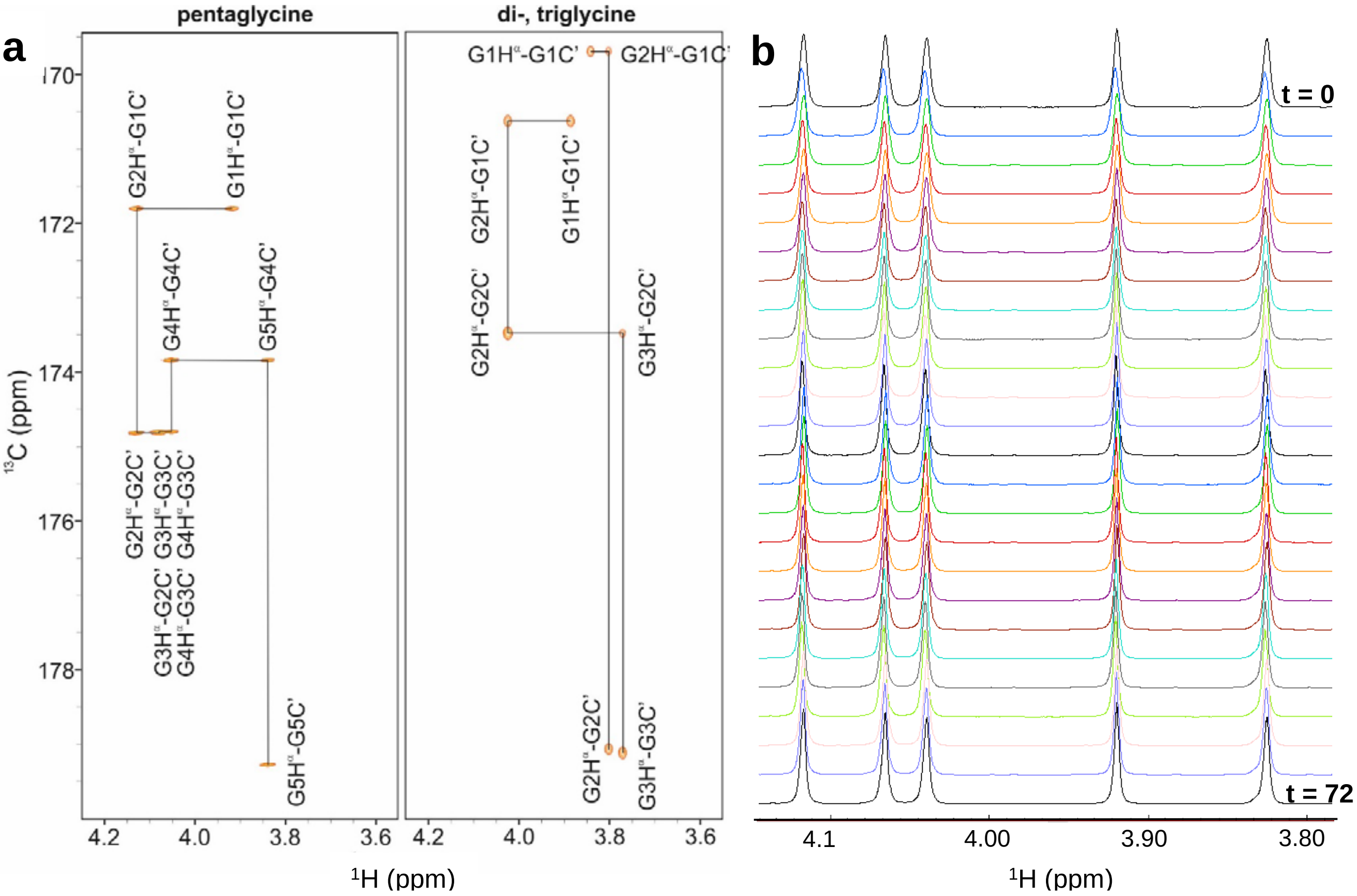

Supplementary Figure S3. Pentaglycine NMR. (a) Identification of substrate (left) and product peaks (right) using a ^{1}H, ^{13}C HMBC spectrum, which gives correlations between protons and carbons separated by two or three bonds. Correlations are annotated. In pentaglycine, all glycines are linked whereas in the product connections are observed between three and two glycines, indicating that LytU pentaglycine hydrolysis products are a tri- and a diglycine. **(b)** Pentaglycine (1 mM) stability under typical reaction conditions (PBS, pH 7.2, 37 °C) without enzyme. Spectra were acquired at three hour intervals for 72 h.

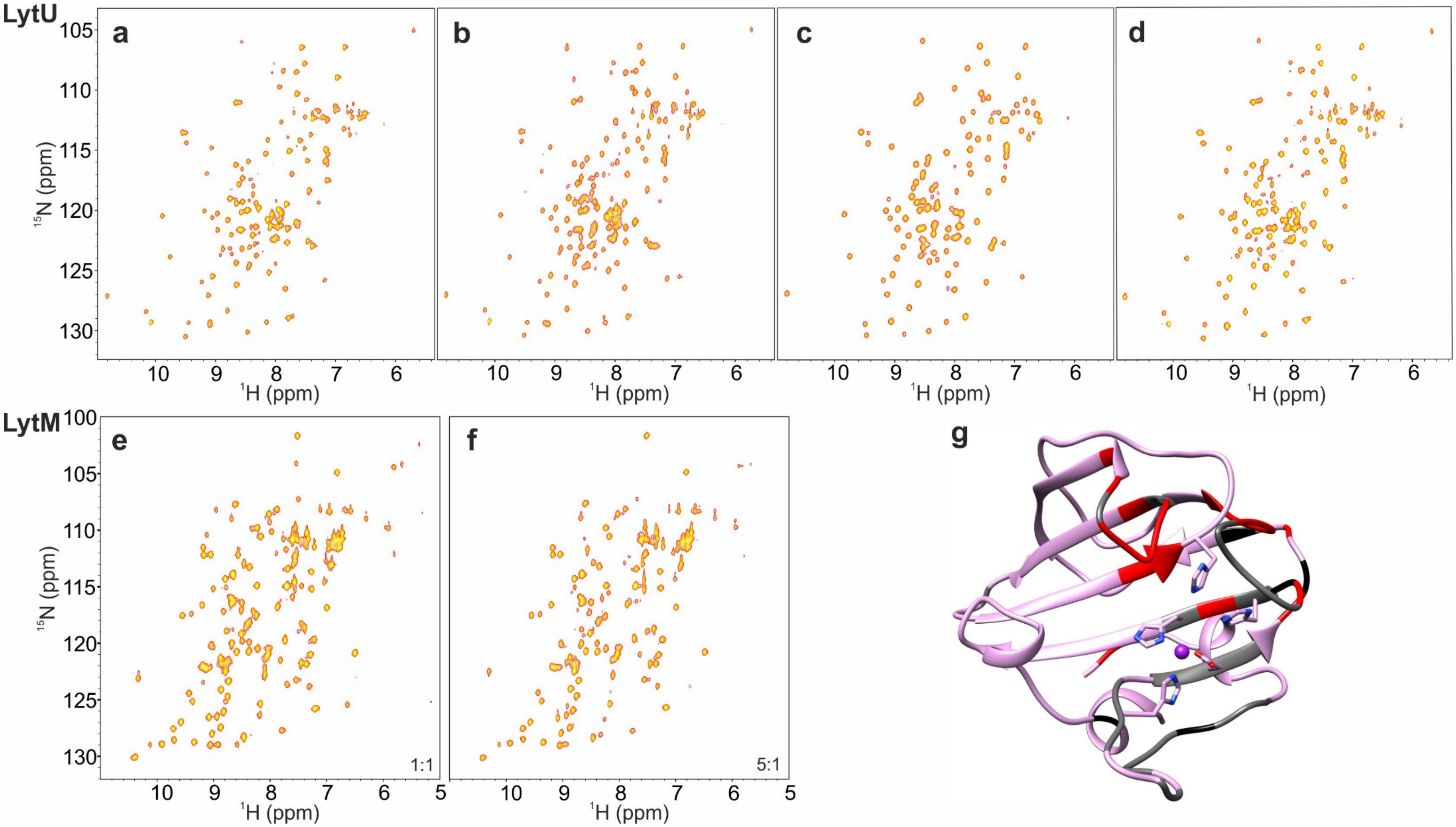

Supplementary Figure S4. ^{1}H, ^{15}N HSQC spectra. Top row: LytU constructs, **(a)** LytU(47-192) N148A mutant, **(b)** LytU(47-192) Δ151 mutant, **(c)** LytU(70-192) construct, and **(d)** LytU(49-192) for comparison. Chemical shift assignments of LytU can be found in Raulinaitis et al. (2017)[1]. **Bottom row:** Comparison of LytM zinc states, **(e)** LytM in 1:1 Zn to protein state, **(f)** LytM in 5:1 excess zinc state, and **(g)** depiction (in red) of LytM residues which undergo chemical shift perturbations upon addition of zinc. Residues not observed in the 1:1 LytM ^{1}H, ^{15}N HSQC spectrum are colored in grey and prolines are in black.

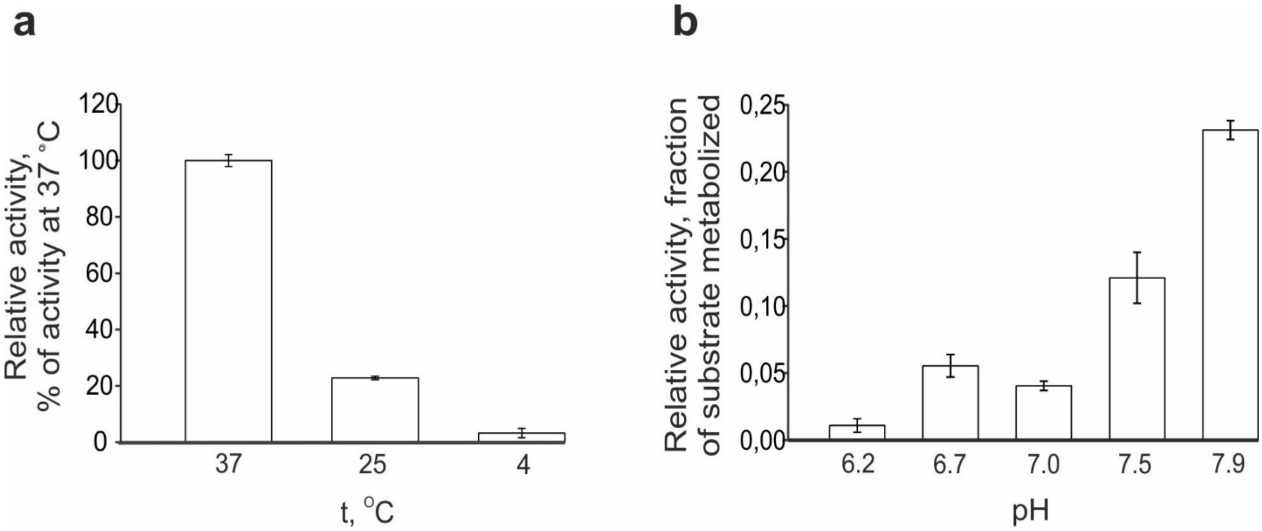

Supplementary Figure S5. LytU pentaglycine cleavage activity. (a) LytU-Ile catalytic domain (49-192) activity of pentaglycine cleavage at different temperatures in PBS (pH 7.3) and **(b)** at different pH values in phosphate buffer (37 °C). The reaction duration was 60 h. Values are presented as mean of at least two independent measurements and error bars indicate standard error (SEM).

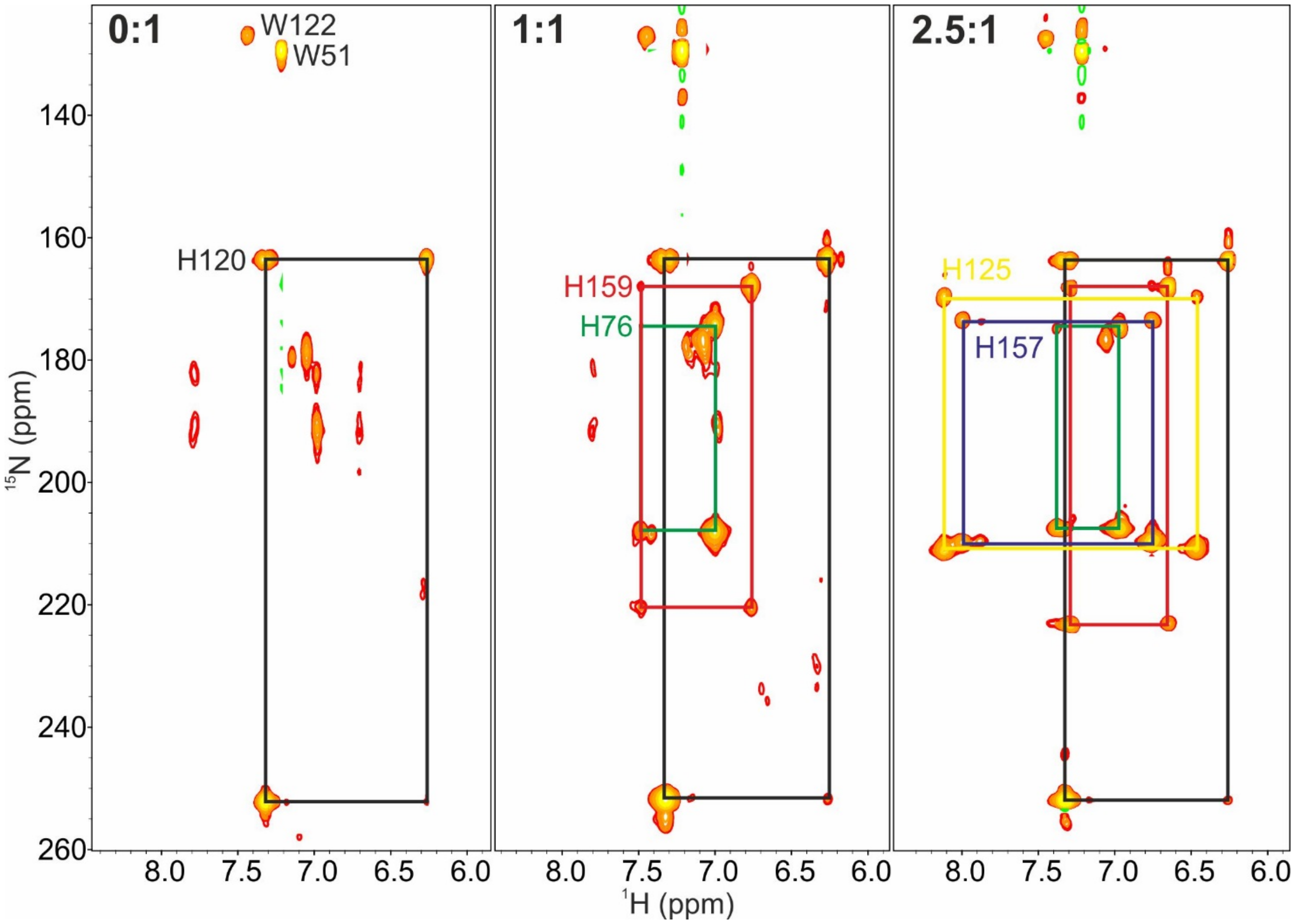

Supplementary Figure S6. ^{1}H, ^{15}N histidine-region HMBC spectra of apo, 1Zn and 2Zn LytU at pH 6.5. In the apo form spectrum only one set of sharp signals is observed. This signal pattern originates from an $H^{\varepsilon2}$ protonation state of H120. Structurally, H120 is located in the substrate groove, at the end opposite to the catalytic site, and is not involved in zinc coordination. Upon addition of one equivalent of zinc, signals for H76 and H159 appear. H76 is $H^{\delta1}$-protonated. Assignment of H159 tautomeric state is ambiguous. The distinct ^{15}N chemical shifts of ~210-220 ppm are characteristic for zinc-coordinating ^{15}N nuclei. Addition of a second equivalent of zinc stabilizes H125 and H157 to the $H^{\delta1}$-protonation state. Again, the low-field ^{15}N chemical shifts match those of zinc-coordinated histidines, indicating that the catalytic histidines ligate the second zinc. Zinc coordination was also referred from $C^{\delta2}$, $C^{\varepsilon1}$ chemical shifts of histidine ring, and found consistent with the results obtained from the ^{1}H, ^{15}N HMBC spectra. This analysis gives a $H^{\varepsilon2}$-protonation state for H159. The remaining histidine 69 was not present in the HMBC. This histidine demonstrated linear pH-dependent chemical shift changes over the full range of pH in all titration series indicating that in none of the zinc states is H69-ligated or hydrogen bonded. The observed tautomeric states correspond to those reported for LytM and lysostaphin.

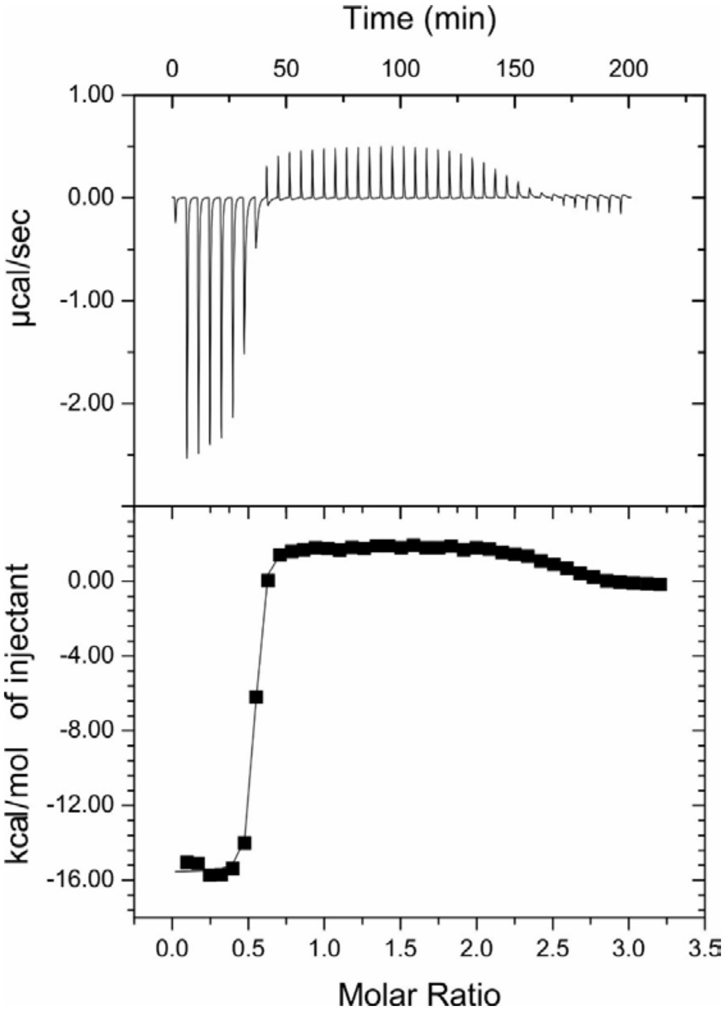

Supplementary Figure S7. ITC titration of LytU with $ZnCl_2$. Upper panel: Typical heat burst curve pattern during 50 µM LytU catalytic domain titration with 800 µM zinc (II) chloride solution in cacodylate buffer at 35 °C. Lower panel: Nonlinear least-square analysis of the data. The binding isotherm was obtained using a two-site model.

Supplementary Table S3. Thermodynamic parameters of zinc binding. Binding of the first ion is enthalpy driven with partial entropy compensation, whereas binding of the second ion is entropy driven. The entropy term of the first ion binding is likely to arise from an overall protein stabilization. Values are provided as means of two or more independent measurements with standard error (SEM). Notably, the exceptional affinity of first zinc binding undermined the efforts for ion removal as the apo form of LytU was apparently partially able to replenish its activity before titration by taking up trace amounts of metal ions during the course of sample handling. This could not be rationally prevented due to the sensitivity of binding and risks of introducing metal chelating agents into the system (e.g. from extra washing steps). Consequently, the stoichiometry of zinc binding was distorted ($N_1 < 1$, $N_2 < 2$). This was, however, of little significance, as stoichiometry was proven by NMR spectroscopy. Moreover, considering the amount of protein contained in the cell and the relatively high Zn^{2+} concentration in the syringe, difference could not affect other thermodynamic parameters.

Protein	Site	K_d (nM)	ΔH (kcal mol $^{-1}$)	TΔS (kcal mol^{-1})	N
LytU-Ile	First	0.26 ± 0.06	–15.1 ± 0.5	–1.6 ± 0.3	0.42 ± 0.09
	Second	323.5 ± 13.5	1.9 ± 0.1	11.1 ± 0.1	2.02 ± 0.07
LytU-Lys	First	0.22 ± 0.09	–14.3 ± 0.2	–0.6 ± 0.1	0.24 ± 0.02
	Second	485.5 ± 194.5	2.8 ± 0.1	11.7 ± 0.2	1.54 ± 0.07

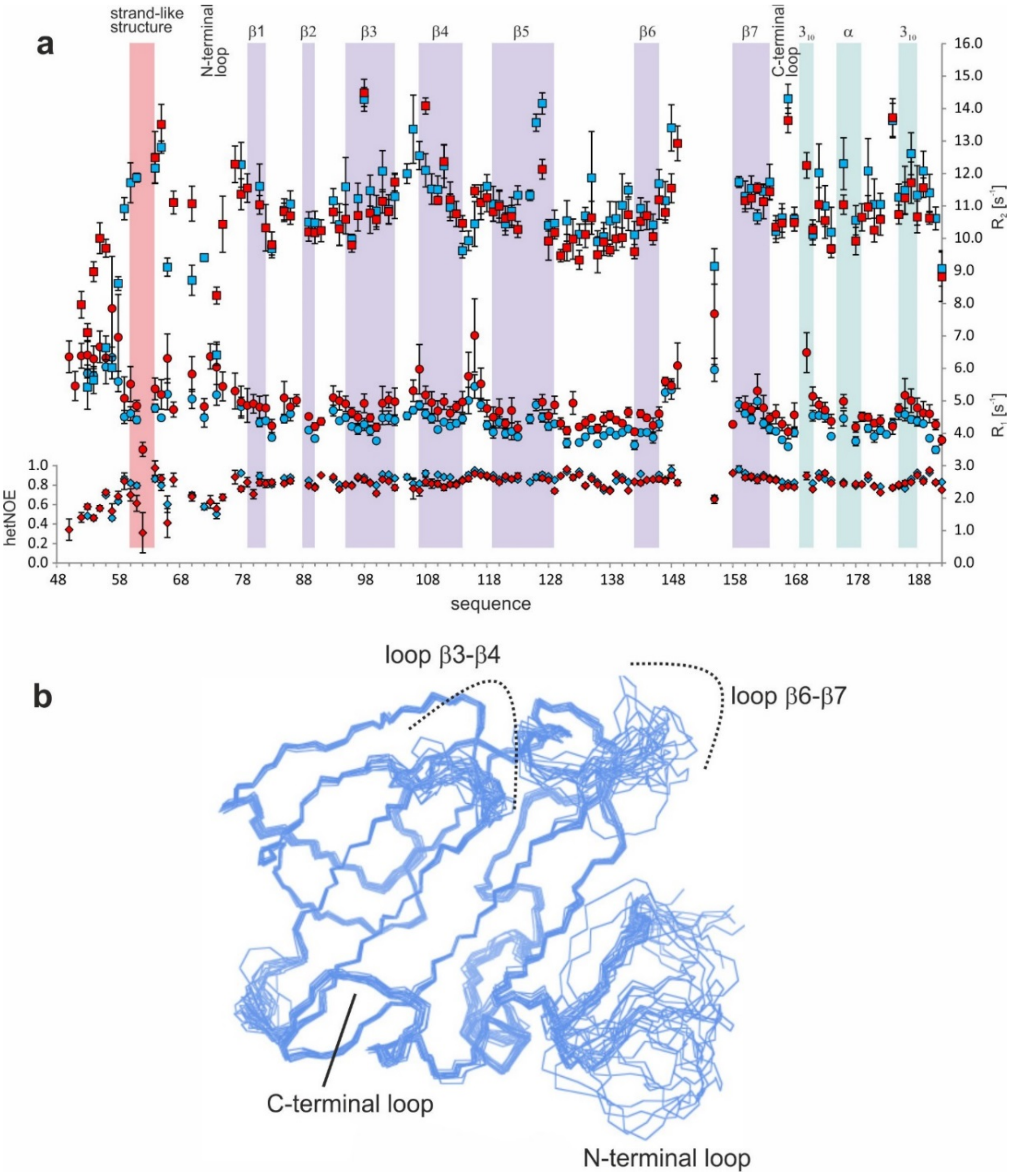

Supplementary Figure S8. One and two zinc-bound LytU ^{15}N relaxation data. **(a)** Heteronuclear NOE data is represented with diamonds, R_1 data with circles and R_2 data with squares. All one-zinc data are shown in blue and two-zinc data in red. Secondary structure elements are shown to simplify navigation within the structure shown below **(b)**. The full soluble part (residues 26-192) of recombinant LytU displayed a relatively poor ^{1}H, ^{15}N HSQC, which originated from the partially unstructured N-terminal region coexisting with a structured domain. It became evident during structure determination that the predicted unstructured region encompasses a yet longer stretch from the N-terminus. For residues 49-57 only intraresidual and sequential NOE signals were observed, indicating lack of a persistent structure. ^{15}N relaxation data indicates high degree of flexibility for residues in this region, i.e. significantly lower than average hetNOE and R_2 values and higher than average R_1 values. Structural dispersion within the structural ensembles present in the N-terminal and the β3-β4 loop is attributed to enhanced flexibility in the ps-ns time range and that of the β6-β7 loop to chemical or conformational exchange in the µs-ms time scale. We note that consistently with LytU, several amide peaks are broadened or missing from the β3-β4 and β6-β7 loops in the LytM ^{1}H, ^{15}N HSQC (Supplementary Fig. S2), suggesting the presence of conformational or chemical exchange. This implies a broader spread of loop conformations in the solution state for LytM, akin to that in LytU.

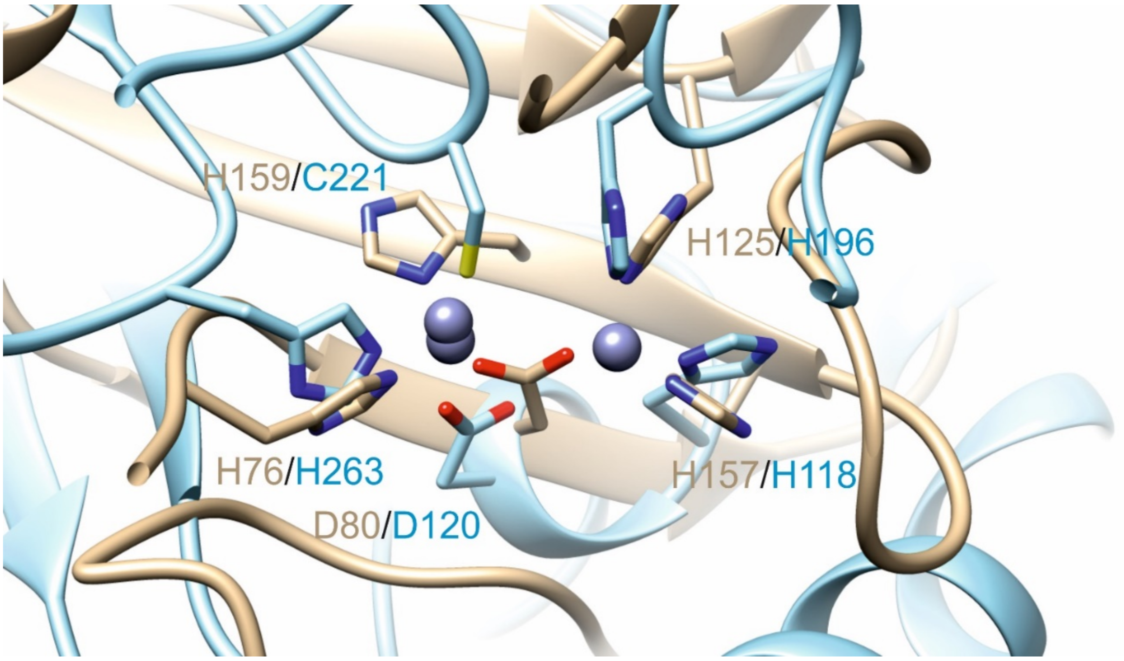

Supplementary Figure S9. Overlay of LytU and CphA catalytic site zinc-coordinating residues. LytU is shown in brown and CphA in blue.

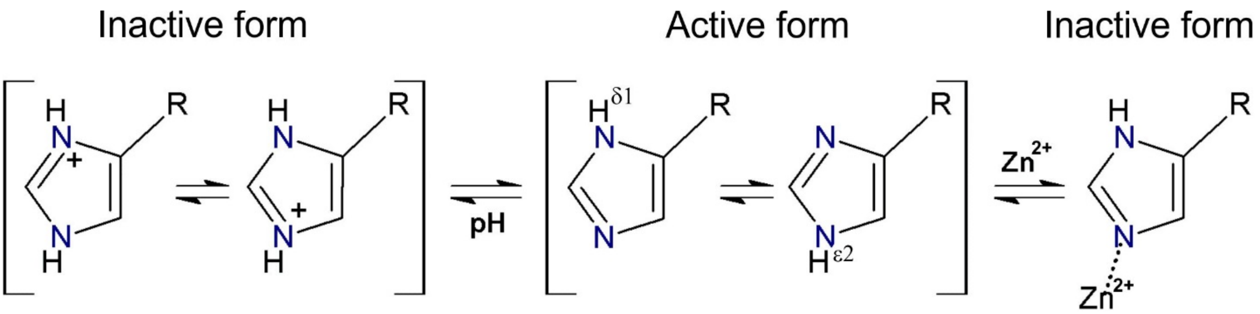

Supplementary Figure S10. Inhibition of LytU catalytic histidines. Tautomeric states of H125 and H157 in active and inhibited by pH or zinc forms.

References

1 Raulinaitis, V., Tossavainen, H., Aitio, O., Seppala, R. & Permi, P. 1H, 13C and 15N resonance assignments of the new lysostaphin family endopeptidase catalytic domain from Staphylococcus aureus. *Biomol NMR Assign* **11**, 69-73 (2017).
